# Revealing the Stabilization Mechanism of Electron-Enriched PtNiCo Catalysts in Practical Direct Methanol Fuel Cells

**DOI:** 10.1021/acscentsci.5c01144

**Published:** 2025-07-30

**Authors:** Min Chen, Yichi Guan, Zhengpei Miao, Shuo Zhang, Chunxia Wu, Yu Zhou, Hongxian Luo, Daoxiong Wu, Ruisong Li, Junming Luo, Xinlong Tian

**Affiliations:** School of Chemistry and Chemical Engineering, School of Marine Science and Engineering, State Key Laboratory of Tropic Ocean Engineering Materials and Materials Evaluation, 74629 Hainan University, Haikou 570228, China

## Abstract

The rational design of Pt-based alloy catalysts with dual resistance to CO poisoning and metal leaching, enabled by interfacial electronic modulation, remains a critical challenge for practical direct methanol fuel cells (DMFCs). Here, we report a highly stable catalyst comprising electron-enriched TiN-meditated PtNiCo (denoted as e-PtNiCo) for DMFCs, demonstrating stabilization mechanisms rooted in enhanced Pt-CO antibonding interactions and strengthened Pt–Co/Ni chemical bonds. The e-PtNiCo catalyst exhibits a voltage decay of 9.6% at 100 mA cm^–2^ over 50 h under practical DMFC operating conditions—a 4-fold improvement compared with the benchmarked PtNiCo (37.7%). Density functional theory calculations and post-mortem elemental analysis reveal that the developed catalysts possess tailored *CO adsorption energetics (−1.62 eV vs −1.27 eV for carbon-supported counterparts) and a 2-fold reduction in Ni/Co dissolution, governed by robust metal–support electronic coupling. This work establishes a mechanistic framework linking support-induced electronic effects to the stability of Pt-based alloys, offering a generalizable strategy for designing structurally durable, high-performance electrocatalysts in energy conversion technologies.

## Graphical abstract

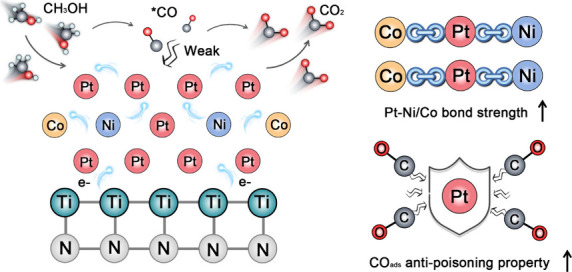

## Introduction

Direct methanol fuel cells (DMFCs) are promising candidates for portable power systems and micropower generation applications, leveraging their facile fuel logistics, compact architecture, and environmental compatibility.^1,2^ Over recent decades, substantial progress has been made in advancing the understanding of the interplay between the composition, dimensions, and morphology of Pt-based methanol oxidation reaction (MOR) electrocatalysts and their activity.^3–7^ However, prior studies have predominantly focused on performance metrics derived from an idealized three-electrode cell (static potential, 25 °C, and low methanol concentrations <1 M), while CO poisoning mechanisms and long-term durability under practical or simulated DMFC conditions remain underexplored.^8,9^ A critical challenge arises in membrane electrode assembly (MEA) systems, where elevated methanol concentrations (2–5 M) exacerbate the accumulation of adsorbed CO intermediates due to sluggish oxidative removal kinetics, thereby imposing stringent demands on MOR catalysts to achieve robust CO tolerance.^10,11^ Furthermore, transition-metal ions (e.g., Ni, Co, Ru) in Pt-based alloys inevitably undergo dissolution under real fuel cell working conditions (0.5–0.8 V, 70–90 °C, and acid microenvironment), which not only degrades catalytic performance but also accelerates the degradation of Nafion membranes and ionomers in the catalyst layer, increasing electrode resistance.^12–14^ These interdependent issues create a critical performance gap, that is, the catalysts that demonstrate exceptional activity in three-electrode configurations often exhibit rapid decay in operational DMFCs.^15^

To address these challenges, recent advances in catalyst engineering propose that incorporating heteroatoms (e.g., La, P, etc.) into Pt matrices to form atomically ordered intermetallic phases could enhance the Pt–M chemical bond strength and optimize CO intermediate adsorption energetics, thereby improving the DMFC performance.^16–21^ However, the synthesis of such ordered Pt-based alloys typical requires high-temperature annealing (>450 °C), which promotes irreversible nanoparticle sintering and compositional heterogeneity, ultimately compromising Pt mass activity and utilization efficiency.^17,22^ Parallel efforts have focused on leveraging interfacial interactions between Pt-based catalysts and conductive supports (i.e., metal oxides, carbides) to modulate the *d*-orbital center—a critical descriptor of CO adsorption strength.^23–27^ For instance, heterogeneous interfaces such as Pt–O–M (M = Ce,^28–30^ Sn^31^) or Pt–C–M (M = Ti^32^) induce localized charge density redistribution, weakening CO binding while enhancing MOR performance. Despite these advances, the operational longevity of Pt-based catalysts remains limited, a challenge largely attributed to interfacial degradation under harsh electrochemical conditions. More critically, the atomic scale stabilization mechanisms enabling synergistic resistance to CO poisoning and metal dissolution, and simultaneously preserving the intrinsic MOR activity, remain insufficiently elucidated.

Herein, we elucidate the stabilization mechanisms enabled by integrating an electron-enriched TiN scaffold into the PtNiCo catalyst, achieving concurrent suppression of CO antipoisoning and transition metal leaching. Concerted experimental and theoretical analyses reveal that the interfacial electron transfer (3.51 e) optimally downshifts the *d*-band center of Pt atoms, reducing the CO adsorption free energy on PtNiCo from −1.62 eV to −1.27 eV. Atomic simulations reveal that this electron-enriched environment strengthens the chemical bond in Pt–Ni and Pt–Co configurations, suppressing dissolution rates of Ni (2.25-fold) and Co (3.35-fold), respectively, during the accelerated durability test. When evaluated in DMFCs, the e-PtNiCo catalysts exhibit exceptional operational stability, retaining 90.4% of the initial voltage and 89.3% of the peak power density after a constant-current operation at 100 mA cm^–2^ for 50 h, underscoring the significant potential for practical application in DMFCs.

## Results and Discussion

### Synthesis and Characterization of e-PtNiCo

The preparation of the e-PtNiCo catalysts is illustrated in Figure 1a. In brief, one-dimensional PtNiCo alloy nanowires (NWs) were prepared through a simple one-pot oil phase synthesis method at 180 °C for 2 h. The element ratio of Pt, Ni, and Co in the obtained PtNiCo alloy NWs was determined near 2:1:1 through inductively coupled plasma atomic emission spectroscopy (ICP-MS) (Table S1). The PtNiCo alloy NWs display a uniform morphology, with a diameter of ∼18.8 nm, as determined by counting 100 randomly selected NWs (Figures 1b and S1). The PtNiCo alloy NWs were immobilized onto the prepared one-dimensional TiN nanotubes (Figures 1c and S2) to form e-PtNiCo. For comparison, carbon-supported PtNiCo alloy NWs (i.e., PtNiCo) were also synthesized using a similar method, replacing TiN with carbon. These PtNiCo alloy NWs were uniformly dispersed on the TiN nanotubes without discernible alterations in the morphology and nanostructure (Figures 1d and S3). The presence of PtNiCo and TiN in e-PtNiCo confirms the successful anchoring of PtNiCo to the TiN (Figure S4). The measured lattice fringes exhibited spacings of 0.225 and 0.247 nm in Lines I and II, respectively, corresponding to the Pt (1 1 1) and TiN (1 1 1) facets (Figures 1e and S5). The corresponding fast Fourier transform pattern revealed a hexagonal phase, with the (1 1 1) and (2 0 0) facets aligning with the satellite peaks characteristic of Pt (Figure 1f inset). The line-scanning analysis of e-PtNiCo demonstrates that both Pt, Ni and Co elements are dispersed uniformly across the whole wires, with the Pt/Ni/Co atomic ratio near 2.9/1/1.7 by energy-dispersive X-ray spectroscopy (EDS) (Figures 1g and S6).

**Figure 1:**
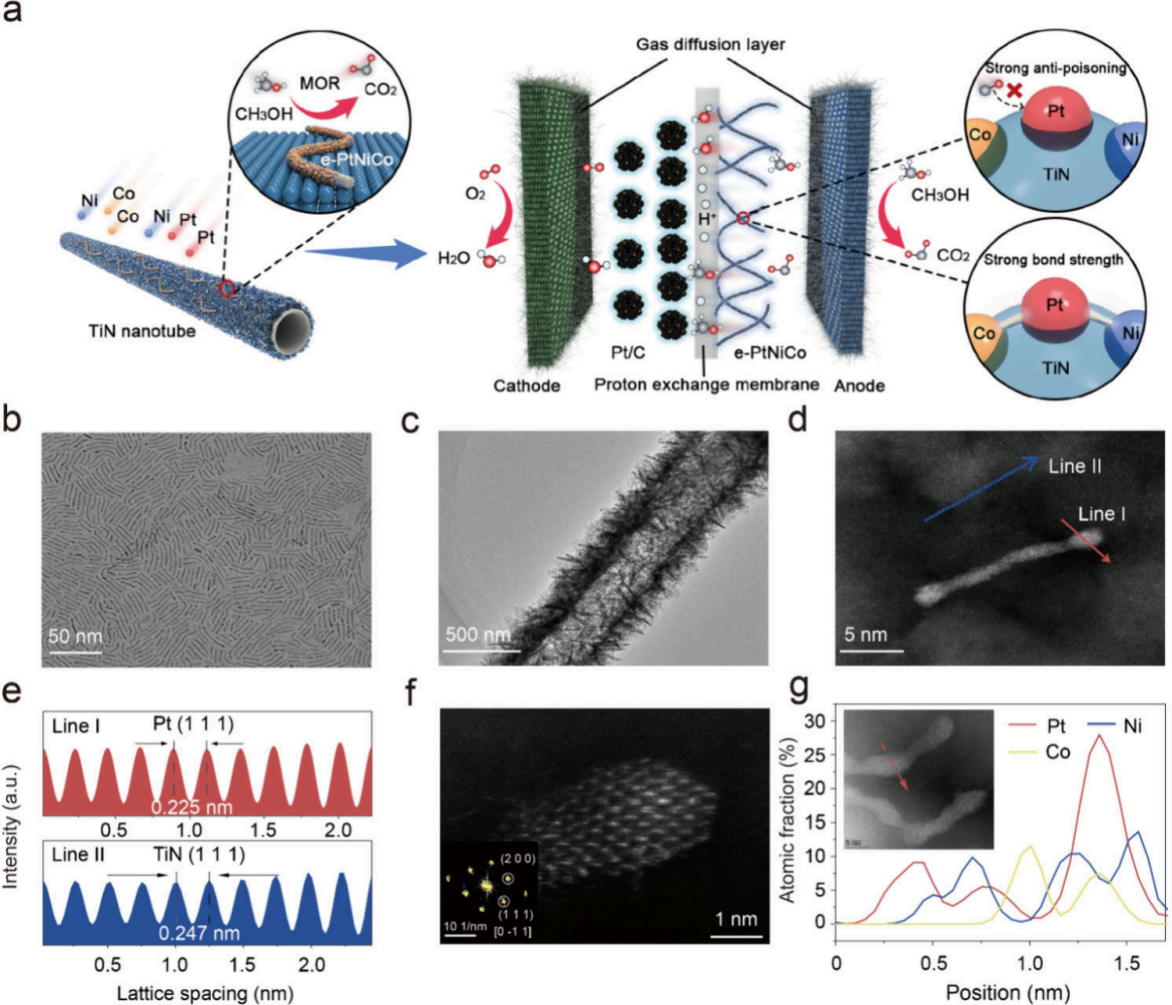
Schematics of the synthesis process and characterization of e-PtNiCo. (a) Schematic illustration for the preparation process of e-PtNiCo catalyst. TEM images of (b) PtNiCo NWs, (c) TiN nanotubes, and (d) e-PtNiCo. (e) The corresponding lattice stripe spacing image from the Line I and II region. (f) HAADF-STEM image of e-PtNiCo. Inset: corresponding FFT pattern. (g) STEM-EDS line-scanning profile (upper right) of single PtNiCo NWs. Inset: the studied PtNiCo NWs and the line-scanning analysis at the red arrow.

### Spectroscopic Characterizations

X-ray photoelectron spectroscopy (XPS) was used to investigate the surface chemical states of e-PtNiCo and the electron transfer interactions among its constituent elements. The binding energies of Pt^0^ 4f_7/2_, Ni^0^ 2p_3/2_, and Co^0^ 2p_3/2_ in e-PtNiCo (71.03, 855.93, and 780.76 eV) shift to lower values compared to those in PtNiCo (71.28, 856.23, and 780.98 eV) and Pt/C (71.60 eV) (Figures S7a–c). In contrast, the Ti 2p_3/2_ binding energy in e-PtNiCo is positively shifted by 0.48 eV compared to TiN (Figure S7d). This shift reflects substantial electron transfer of TiN to the PtNiCo alloy, altering the electronic environment of the active metal sites (Table S2). XPS valence band spectroscopy measurements were conducted to determine the valence band center (i.e., the *d*-band center) of these catalysts.^33–36^ The Pt *d*-band center for e-PtNiCo (−3.64 eV) shifts downward compared to PtNiCo (−3.55 eV) and Pt/C (−3.41 eV), which is expected to reduce the poisoning of CO intermediates for the e-PtNiCo catalyst (Figure S8).

X-ray absorption near-edge structure (XANES) and extended X-ray absorption fine structure (EXAFS) revealed additional information about the valence states and coordination environments of Pt, Ni, and Co as well as the influence of the electronic metal–support interaction on Pt–Pt, Pt–Ni, and Pt–Co bond lengths. The valence state of Pt was inferred from the white line (WL) intensity in the normalized Pt L_3_-edge XANES spectra (Figure 2a). The reduced WL intensity observed for e-PtNiCo (approximately 11,567 eV), corresponding to the 2p_3/2_ to empty 5d orbital transition, indicates a less oxidized state of Pt. In parallel, the WL intensities in the Ni K-edge and Co K-edge XANES spectra of e-PtNiCo and PtNiCo followed the trend of Ni foil (Co foil) < e-PtNiCo < PtNiCo (Figures 2b and 2c). These observations collectively suggest that TiN facilitates a substantial and stable electron supply to PtNiCo, modulating its local electronic environment and promoting the formation of an electron-rich state. The Pt L_3_-edge EXAFS spectra of the samples reveal that the bond lengths of Pt–Pt, Pt–Ni, and Pt–Co in e-PtNiCo are notably shorter than that in PtNiCo (Figures 2d and S9 and Table S3). A similar trend was observed in the Ni K-edge (Figure 2e) and Co K-edge EXAFS spectra (Figure 2f); i.e., Ni–Pt and Co–Pt peaks of e-PtNiCo shifted to lower values compared with those of PtNiCo. This can clearly be attributed to the fact that PtNiCo in the electron-enriched state effectively enhances the electronic interactions of Pt–Pt, Pt–Ni, and Pt–Co bonds, thereby reducing their interatomic distances (Figures 2g and 2h). Furthermore, the wavelet transformation of the χ­(*k*) spectra for e-PtNiCo and PtNiCo reveals only one merged scattering path signal for Pt–Pt/Ni/Co bonds located at (χ­(*k*), χ­(*R*)) values of (9.1, 2.6) and (10.2, 2.7), respectively (Figure S10). The position of the Pt–Pt/Ni/Co bond in e-PtNiCo is slightly shifted toward lower *R* and *k*-ranges compared to PtNiCo, implying a shorter Pt–Pt/Ni/Co bond length due to the stronger electron interaction between Pt–Pt/Ni/Co induces greater compressive strain in the Pt–Pt/Ni/Co bond.

**Figure 2:**
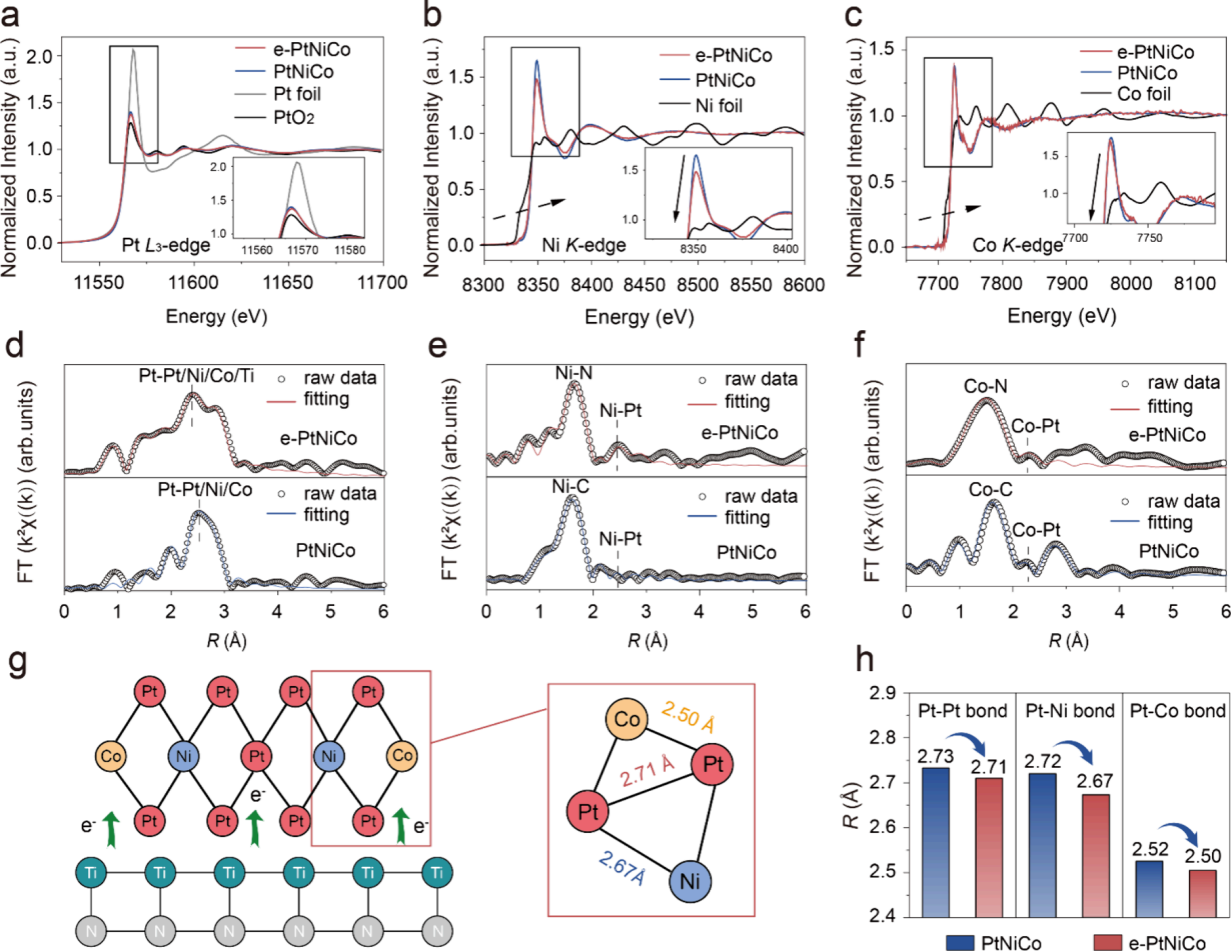
Spectroscopic characterizations of e-PtNiCo. XANEs spectra of (a) Pt L_3_-edge XANEs spectra, (b) Ni K-edge XANEs spectra, and (c) Co K-edge XANEs spectra of e-PtNiCo, PtNiCo and standard samples. (d) Pt L_3_-edge *k* ^3^-weighted FT-EXAFS spectra, (e) Ni K-edge *k* ^3^-weighted FT-EXAFS spectra, and (f) Co K-edge *k* ^3^-weighted FT-EXAFS spectra of e-PtNiCo and PtNiCo. (g) The model of e-PtNiCo metallic bond length. (h) Comparison of Pt–Pt, Pt–Ni, and Pt–Co bond lengths of e-PtNiCo and PtNiCo.

### Electrocatalytic Performance of e-PtNiCo

The electrochemical performance of the studied catalysts for MOR was evaluated in a gastight standard electrolyte comprising N_2_-saturated 0.1 M HClO_4_ and 0.5 M CH_3_OH solution after being activated and cleaned in 0.1 M HClO_4_ until stable cyclic voltammetry (CV) (Figure S11). The electrochemically active surface areas (ECSAs) of e-PtNiCo, PtNiCo, and Pt/C were determined based on charge measurements from the hydrogen underpotential deposition (H_UPD_) region, yielding values of 49.0 m^2^ g^–1^, 48.4 m^2^ g^–1^, and 48.1 m^2^ g^–1^, respectively. The CO-stripping method was also used to assess the ECSAs, with results of 59.4 m^2^ g^–1^, 58.1 m^2^ g^–1^, and 57.8 m^2^ g^–1^, respectively (Figure S12 and Table S4). The e-PtNiCo catalyst displays the lowest onset oxidation potential of 0.50 V vs the reversible hydrogen electrode (RHE) and the highest mass activity (MA) of 1.73 A mg_Pt_^–1^, outperforming PtNiCo (0.56 V vs RHE, 0.79 A mg_Pt_^–1^) and Pt/C (0.58 V vs RHE, 0.28 A mg_Pt_^–1^) (Figures 3a and 3b). Meanwhile, the specific activity (SA) of e-PtNiCo (3.54 mA cm^–2^) exhibited a higher value than those of PtNiCo (1.63 mA cm^–2^), Pt/C (0.58 mA cm^–2^), and other recently reported noncarbon catalysts (Figure S13 and Table S5). In addition, the performance of TiN-supported PtNi, PtCo, and Pt catalysts was further investigated, and it was found that e-PtNiCo outperforms these catalysts (Figure S14). The *I*_f_/*I*_b_ value for e-PtNiCo is 1.17, whereas those for PtNiCo and Pt/C are approximately 0.92 and 1.02, respectively. This indicates that the e-PtNiCo catalyst exhibits a better CO-poisoning tolerance. The CO-stripping experiment was further applied to assess the antipoisoning ability of adsorbed CO intermediates. As depicted in Figure 3c, the onset oxidation potential of CO_ads_ on e-PtNiCo (0.615 V vs RHE) is significantly lower than that on PtNiCo (0.742 V vs RHE) and Pt/C (0.750 V vs RHE) catalysts, indicating a favorable anti-CO poisoning ability exhibited by the e-PtNiCo catalyst.

**Figure 3:**
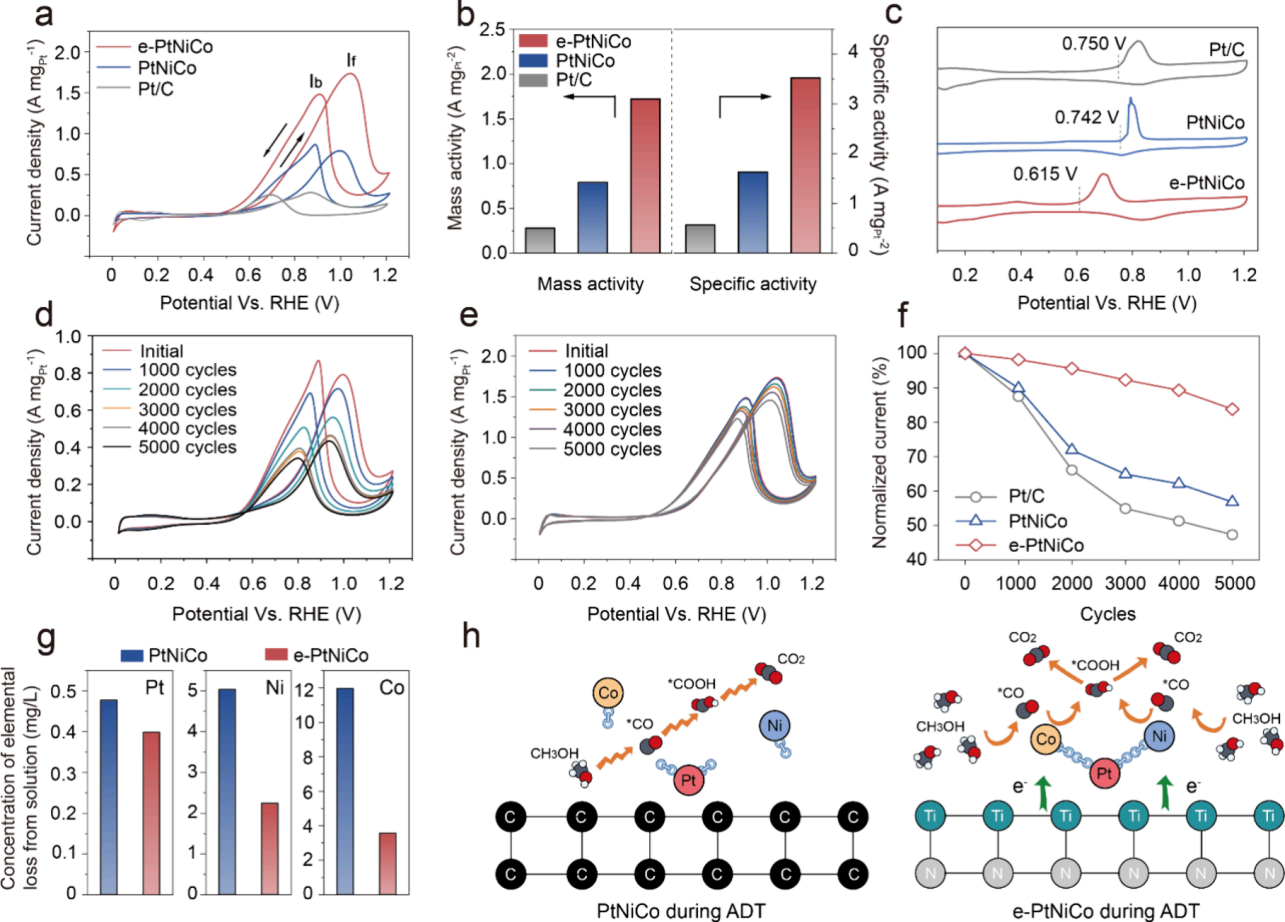
Electrocatalytic performance of the prepared and commercial catalysts. (a) MA of e-PtNiCo and reference samples in 0.1 M HClO_4_ containing 0.5 M CH_3_OH. (b) The activity histogram of the studied catalysts. (c) CO stripping curves of e-PtNiCo and reference samples. The evolution of CVs for (d) PtNiCo and (e) e-PtNiCo with continuous 5000 ADT. (f) The plots of peak current change versus the cycle number for MOR on e-PtNiCo and reference samples. (g) Dissolving rate of Pt, Ni, and Co metals for e-PtNiCo and PtNiCo after 5000 ADT. (h) Schematic representation of methanol oxidation of e-PtNiCo and PtNiCo during ADT.

The stability of the catalysts was checked using the accelerated durability test (ADT) in a 0.1 M HClO_4_ + 0.5 M CH_3_OH solution. The activity of the e-PtNiCo remained as high as 83.8% after 5000 cycles of ADT, while those of PtNiCo (56.8%) and Pt/C (47.3%) under the same conditions were lower (Figures 3d–f and S15). The morphology of the e-PtNiCo is preserved well and the element ratio of Pt:Ni:Co remains 2:1:1, but the PtNiCo shows structural collapse and aggregation after ADT. The Co and Ni atoms decreased significantly (Figures S16 and S17). The Pt^0^ 4f_7/2_ spectra peaks of e-PtNiCo exhibit a slight positive shift of 0.10 eV, but a larger shift of 0.64 eV for PtNiCo after ADT (Figure S18a,b). Similarly, the Ni^0^ 2p_3/2_ and Co^0^ 2p_3/2_ peaks of e-PtNiCo showed minimal shifts, whereas the intensity of the Ni^0^ 2p_3/2_ peak decreased sharply, and no signal for Co element was detected in PtNiCo (Figure S18c–f). The Ti–N bond and TiN structure remained intact after ADT cycles (Figure S19). The dissolution of Pt, Ni, Co for these catalysts in the electrolyte were examined based on ICP-MS test. The leached masses of Pt, Ni, and Co metals for PtNiCo in the electrolyte are 1.20, 2.25, and 3.35 times higher than e-PtNiCo after 5000 ADT (Figure 3g). Due to the dissolution of these unstable Co and Ni, the electron supply of Pt is reduced, and the Pt *d*-band center of PtNiCo is shifted upward with a large contrast with e-PtNiCo, and the corresponding resistance to poisoning by CO is reduced (Figure S20). These findings collectively suggest that TiN provides a continuous electron supply to PtNiCo, promoting the formation of an electron-enriched state. This not only enhances the CO poisoning resistance of the PtNiCo alloy but also suppresses the dissolution of Ni and Co metals. This dual-modulation enables e-PtNiCo to achieve an efficient and stable MOR (Figure 3h).

### Direct Methanol Fuel Cell Performance and Durability

The e-PtNiCo catalyst was further implemented in the membrane electrode assembly to evaluate its realistic performance and stability as an anode of DMFCs. Polarization curves (*I–V*/*I–P*) reveal that the DMFCs with the e-PtNiCo deliver a maximum power density (*P*_max_) of 107 mW cm^–2^ at 65 °C, surpassing those of PtNiCo (63 mW cm^–2^) and Pt/C (37 mW cm^–2^) (Figure 4a). Furthermore, e-PtNiCo demonstrates a significantly higher and more stable output voltage (OV) when tested at a constant current of 100 mA cm^–2^ at 65 °C in 1 M methanol (Figure 4d). Only a 9.6% loss (loss rate: 0.88 mV h^–1^) in OV is observed after 50 h of operation, in stark contrast to PtNiCo (5.96 mV h^–1^) and Pt/C (6.08 mV h^–1^), which exhibit 37.7% and 41.9% decreases in OV after 25 h, respectively. The *I–V*/*I–P* plots in Figure 4b show that the *P*_max_ of e-PtNiCo decreases by approximately 10.3%, from 107 to 96.3 mW cm^–2^, after 50 h, while PtNiCo and Pt/C show performance reductions of 28.7% and 37.9%, respectively, after 25 h. Notably, the e-PtNiCo catalyst outperforms other catalysts previously reported for DMFCs, in terms of both mass-specific power density and durability (Figure 4e and Table S6). The superior durability of e-PtNiCo is attributed to its excellent resistance to poisoning and metal dissolution, which allows the catalyst to remain structurally stable after constant current tests (Figures 4f and S21).

**Figure 4:**
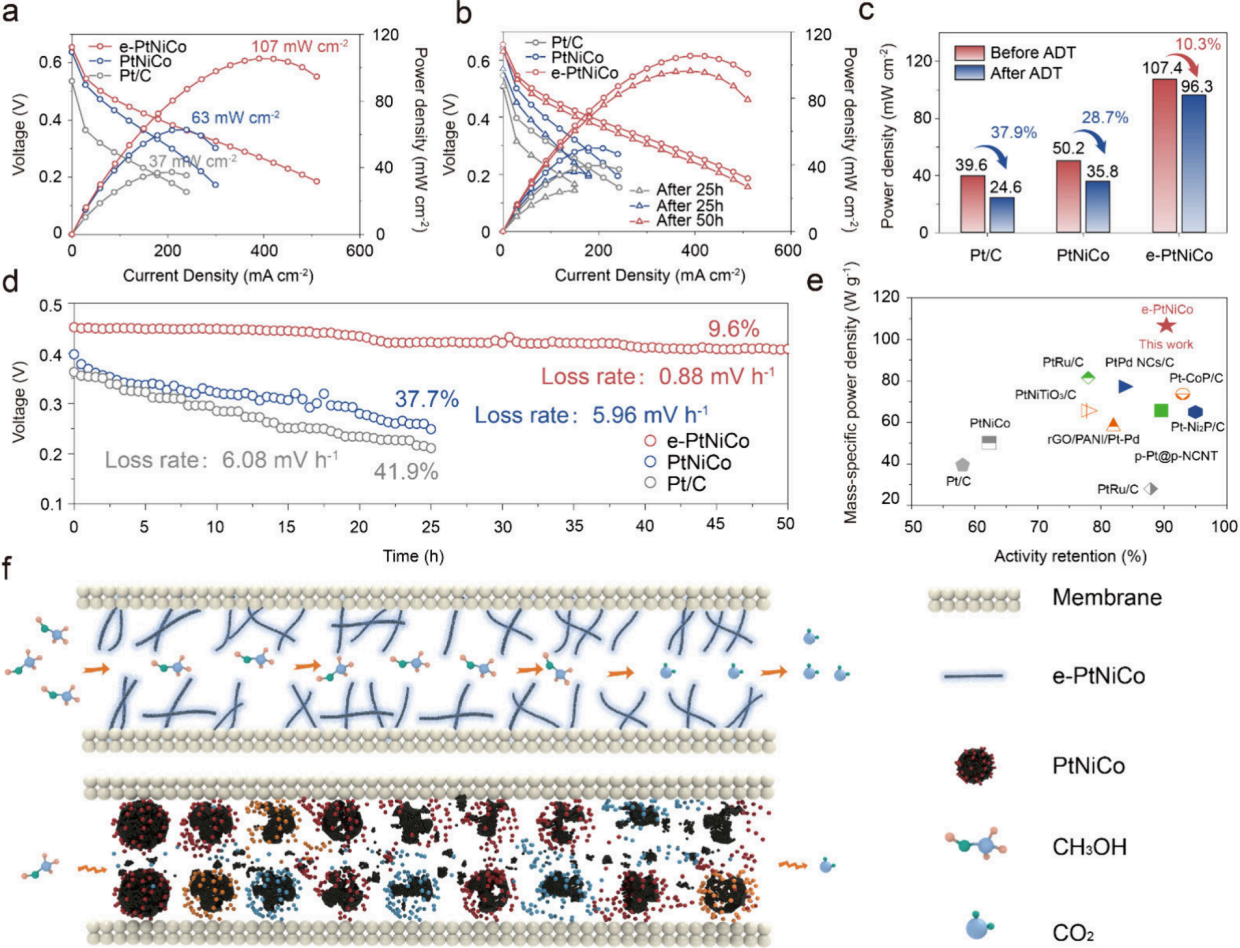
Fuel cell performance of the prepared and commercial catalysts. (a) Fuel cell polarization curves of e-PtNiCo and reference samples. (b) Fuel cell polarization curves and (c) mass-specific power density of e-PtNiCo and reference samples before and after 25 or 50 h. (d) Stability of the DMFCs at 100 mA cm^–2^. Methanol concentration: 1.0 M, cell temperature: 65 °C. (e) Comparison of the mass-specific power density and activity retention of e-PtNiCo and recently reported works. (f) Schematic representation of e-PtNiCo and PtNiCo catalyst layers during fuel cell operation.

### Mechanism Study

Next, we employed density functional theory (DFT) calculations to elucidate the mechanism by which TiN supports enhance the catalytic stability of PtNiCo materials through dual modulation. The crystal structures of the pristine PtNiCo model and the PtNiCo loaded onto TiN (denoted as e-PtNiCo/TiN) model are shown in Figure S22. Significant charge density redistribution occurs at the interface between PtNiCo and TiN, as depicted in Figure 5a. The TiN side exhibits a region of reduced charge density, while the PtNiCo side shows an increased charge density region, indicating charge transfer from TiN to PtNiCo. Bader charge analysis quantifies the charge transfer as 3.51 e, which puts the PtNiCo catalyst into an electron-enriched state. This charge redistribution alters the bonding characteristics of the PtNiCo catalyst. The orbital overlap between the surface atoms of the catalyst and adsorbates leads to bonding orbitals with lower energy levels and antibonding orbitals with higher energy levels. Relative to the PtNiCo catalyst, the *d*-band center of Pt atoms in the electron-enriched PtNiCo catalysts shifts to a deeper energy level, from −2.29 eV to −2.74 eV. According to *d*-band center theory proposed by Noskov et al.,^37^ this shift results in increased electron occupancy in the antibonding orbitals, weakening the bond between the catalyst and reactive species (Figure 5b). This effect is particularly beneficial in the methanol oxidation process, where CO poisoning is a major issue due to the strong binding of CO to Pt-based catalysts. In the e-PtNiCo/TiN system, the Gibbs free energy of CO adsorption decreases from −1.62 eV in PtNiCo to −1.27 eV, indicating a significant reduction in the level of CO poisoning (Figure 5c). The suppression of CO poisoning is attributed to the deeper Pt *d*-band center induced by the electron transfer between TiN and PtNiCo.

**Figure 5:**
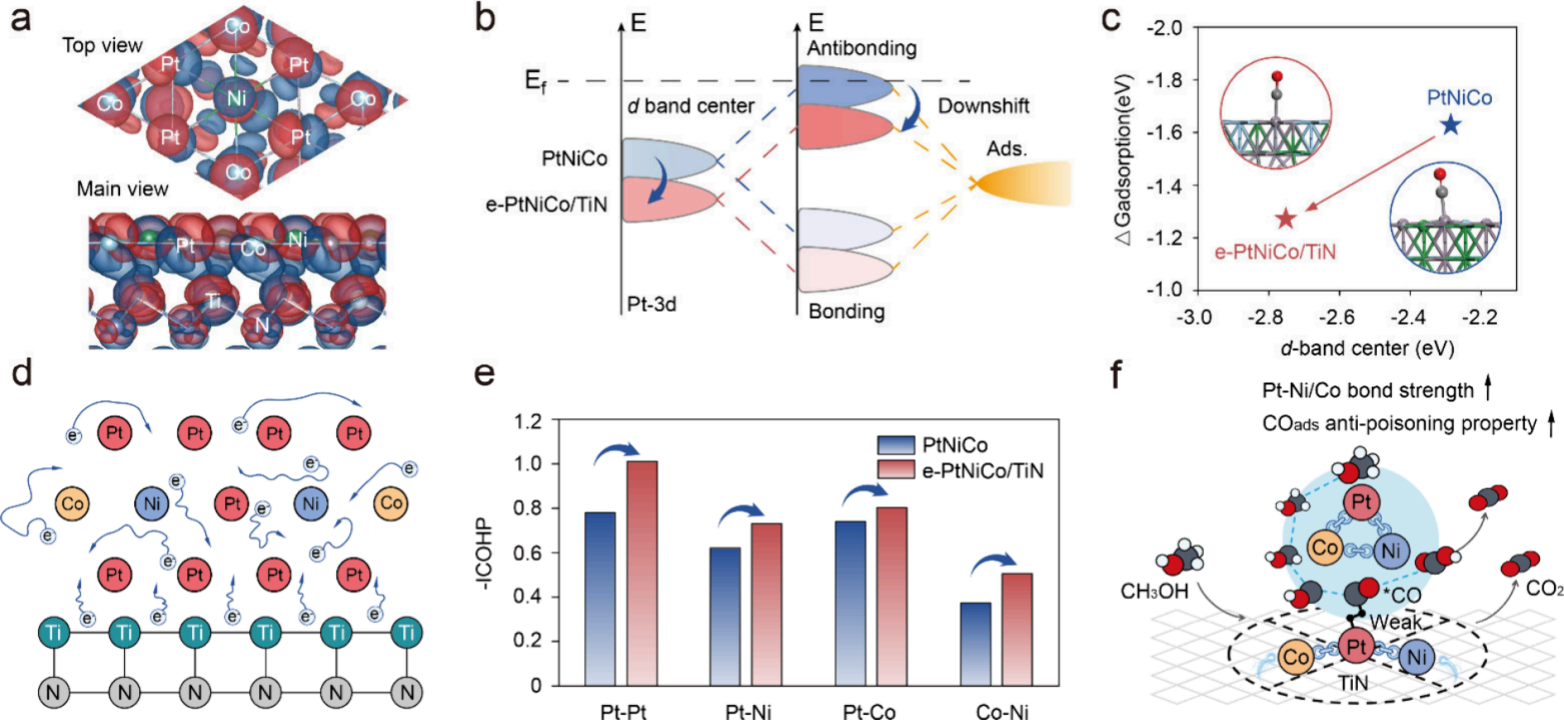
Mechanism study. (a) Differential charge density plot for e-PtNiCo/TiN. Blue and red regions indicate electron accumulation and depletion, respectively. The isosurface value is set to be 0.02 e/bohr^3^. (b) Schematic diagrams illustrating the *d*-band position of e-PtNiCo/TiN. (c) Relationship between *d*-band centers of platinum atoms in PtNiCo and e-PtNiCo/TiN and CO adsorption energy. (d) The charge transfer model for e-PtNiCo/TiN. (e) -ICOHP of Pt–Pt, Pt–Ni, Pt–Co, and Co–Ni bonds in PtNiCo and e-PtNiCo/TiN structures. (f) Dual modulation diagram of PtNiCo on TiN.

There are metallic bonds formed by interactions between nearly free electrons and the atomic nuclei in PtNiCo that consist of metal atoms. The Electron Localization Function (ELF) analysis reveals the presence of nearly free electrons with a localization degree around 0.5 between the metal atomic nuclei (Figure S23). These nearly free electrons mediate the formation of metallic bonds with the nuclei. Moreover, charge transfer leads to an increase in the number of delocalized electrons in PtNiCo. These are expected to further enhance metallic bonding within PtNiCo in e-PtNiCo/TiN, as shown in Figure 5d. The integrated crystal orbital Hamilton population (-ICOHP) value was calculated to analyze bonding strength between key atomic pairs, including Pt–Pt, Pt–Ni, Pt–Co, and Co–Ni pairs, in PtNiCo and e-PtNiCo/TiN (Figure 5e). Among the pristine PtNiCo catalysts, the Co–Ni pair has the smallest -ICOHP value, implying that it is the weakest bond and is the most prone to breaking during catalysis. This is consistent with experimental observations showing higher dissolution of Ni and Co compared to Pt in PtNiCo (Figure 3g). In contrast, the -ICOHP values for all four atom pairs in e-PtNiCo/TiN increased, suggesting strengthened atomic bonding and, thus, effectively suppressing metal dissolution during catalytic reactions. Therefore, the dual modulation of PtNiCo by TiN is reflected in the effective mitigation of CO poisoning as well as the significant inhibition of metal dissolution, see Figure 5f. The combined effect of these two mechanisms results in an e-PtNiCo/TiN catalyst with excellent methanol oxidation stability.

## Conclusions

This work reports the rational design of an electron-enriched PtNiCo catalyst with dual functionality: robust CO poisoning resistance and exceptional resistance to metal leaching. Combined X-ray absorption spectroscopy and DFT calculations elucidate how sustained electron donation from the TiN scaffold induces charge redistribution across the PtNiCo surface, simultaneously strengthening Pt–Ni/Co chemical bonds and stabilizing the alloy against acidic dissolution. The e-PtNiCo system exhibits 2.25-fold lower Ni dissolution and 3.35-fold reduced Co leaching relative to conventional PtNiCo during prolonged methanol oxidation, directly correlating with its superior DMFC durability—retaining 90.4% of the initial voltage after 50 h at 100 mA cm^–2^. Crucially, interfacial electron transfer from TiN lowers the CO adsorption free energy on surface Pt sites from −1.62 eV to −1.27 eV, effectively shifting the *d*-band center away from the Fermi level and achieving a mass-specific power density of 107 W g_Pt_^–1^. This dual-functionality strategy establishes a universally applicable paradigm for Pt-based catalyst engineering, thereby overcoming the long-standing activity–stability dichotomy that impedes practical DMFCs.

## Supplementary Material



## Notes

Chemicals and materials, equipment, experimental section, computational methods, characterization results, and additional data (PDF)
