# Supplementary material for: Revealing the Stabilization Mechanism of Electron-Enriched PtNiCo Catalysts in Practical Direct Methanol Fuel Cells
Source: ACS Cent Sci. 2025 Jul 30;11(10):1862–9. doi: 10.1021/acscentsci.5c01144 (PMC12550620; doi:10.1021/acscentsci.5c01144)
Supplement: Supplementary file 1 [file oc5c01144_si_001.pdf]

Supporting Information

Revealing the Stabilization Mechanism of Electron-Enriched PtNiCo Catalysts in Practical Direct Methanol Fuel Cells

Min Chen[#], Yichi Guan[#], Zhengpei Miao*, Shuo Zhang, Chunxia Wu, Yu Zhou, Hongxian Luo, Daoxiong Wu, Ruisong Li, Junming Luo, Xinlong Tian*

School of Chemistry and Chemical Engineering, School of Marine Science and Engineering, State Key Laboratory of Tropic Ocean Engineering Materials and Materials Evaluation, Hainan University, Haikou 570228, China

[#]*The authors contributed equally to this work.*

*Corresponding author. Email: *zpmiao92@hainanu.edu.cn (Z. Miao); tianxl@hainanu.edu.cn (X. Tian)*

Materials and Methods

Material and Chemicals. Platinum(II) acetylacetonate ($Pt(acac)_2$, 97%), nickel(II) acetylacetonate [$Ni(acac)_2$, 95%], Cobalt(II) acetylacetonate [$Ni(acac)_2$, 95%], molybdenum hexacarbonyl [$Mo(CO)_6$, 98%], hexadecyl trimethyl ammonium bromide (CTAB, 95%), oleylamine (OAm, 80-90%), diphenyl ether (DPE, AR), Titanium (IV) oxysulfate-sulfuric acid hydrate ($TiOSO_4 \cdot xH_2SO_4 \cdot xH_2O$), Ethanol, Ethyl ether and Glycerol were all purchased from Shanghai Macklin Biochemical Co., Ltd. 5 wt.% Nafion solution was bought from Sinopharm Chemical Reagent Co., Ltd. 20 wt.% Pt/C was obtained from Johnson Matthey Co., Ltd. All reagents were used without further purification, and all solutions were freshly prepared with ultrapure water.

Preparation of ultrathin PtNiCo alloy nanowires (NWs). Typically, $Pt(acac)_2$ (10.0 mg), $Ni(acac)_2$ (6.4 mg), $Co(acac)_2$ (8.9 mg), $Mo(CO)_6$ (9.0 mg), and CTAB (110.0 mg) powders were immersed into a mixed solution of OAm (3.0 ml) and DPE (2.0 ml) in a clean round-bottomed flask and ultrasonication for 1 h to get a transparent solution. Then, the mixture was transferred into an oil bath and kept at 180°C for 2 h. After being cooled down to room temperature, the product was collected by centrifugation at a speed of 10,000 rpm for 10 min, washed three times with a cyclohexane/ethanol (1:9, v/v) mixture, and then redispersed in 10 ml cyclohexane for further use.

Preparation of TiN nanosheet assembly hollow tube. In a typical procedure, $TiOSO_4 \cdot xH_2SO_4 \cdot xH_2O$ (1.0 g) was introduced to a solution comprising ethanol (28 ml), ethyl ether (16 ml), and glycerol (16 ml). The mixture was continuously stirred for 1 hour followed by 3 h of sonication to achieve a milky white homogeneous solution. Subsequently, the solution was transferred into a 100 ml Teflon-lined autoclave and maintained at 130°C for 12 h. The resulting white TiO_2 were collected by filtration, washed three times with absolute ethanol,

and then vacuum dried at 60°C overnight. Finally, to produce TiN, the white TiO_2 powder was placed in a program-controlled heating tubular furnace and programmed to heat up to 800°C (from room temperature to 500°C at 5°C min^{-1}; 500-600°C at 2°C min^{-1}; 600-800 at 1°C min^{-1}) under ammonia (NH_3) gas flow (40 sccm) and maintained for 4 h.

Preparation of e-PtNiCo catalyst. 16 mg as-prepared TiN was immersed into 25 ml ethanol and stirred for 0.5 h. Then, a certain amount of PtNiCo NWs solution was slowly added with stirring, followed by ultrasonication for 4 h. The mixture was evaporated at 130°C, and the resulting solid product obtained was collected. Finally, the product was placed in a program-controlled heating tubular furnace and programmed to heat up to 130°C under Ar/H_2 gas (95:5) for 2 h.

Preparation of PtNiCo catalyst. The PtNiCo catalyst were prepared by the same procedure, as the PtNiCo using 16 mg Carbon (Ketjen Black) instead of the above-mentioned 16 mg TiN.

Materials characterization

Scanning electron microscopy (SEM) studies were performed on a Hitachi S4800 microscope. Transmission electron microscopy (TEM) images and high-angle annular dark-field scanning transmission electron microscopy (HAADF-STEM) images were taken from a Thermo Fisher Talos F200X G2 with an operation voltage of 200 kV. The X-ray diffraction (XRD) patterns were collected on a Rigaku Smart-Lab using Cu-Kα radiation with a wavelength of 1.54 Å. Inductively coupled plasma mass spectrometry (ICP-MS) (PerkinElmer, Nexlon 300D) was utilized to measure the loading of Pt, Co, Ni in as-prepared catalysts, as well as their dissolution in the electrolyte after the catalyst was tested by 5000 cyclic voltammograms (CVs).

X-ray photoelectron spectroscopy (XPS) and valence band spectra studies were carried out on a Thermo Scientific K-Alpha. The X-ray absorption spectra (XAS) including X-ray absorption near-edge structure (XANES) and extended X-ray absorption fine structure (EXAFS) of the samples at Pt L_3-edge, Ni K-edge and Co K-edge (collected at beamline BL11B of the Shanghai Synchrotron Radiation Facility, China.) The storage ring was working at the energy of 8.0 GeV with an average electron current of below 99 mA. The acquired EXAFS data were extracted and proceed according to the standard procedures using the Athena (version 0.9.26) module implemented in the FEFIT software packages. The k^2-weighted Fourier transform (FT) of $x(k)$ in R space was obtained over the range of 0-14.0 Å^{-1} by applying a Besse window finction. Wavelet transformation (WT) spectra were also employed using the software package developed by Funke and Chukalina and the Morlet wavelet with k = 10, and σ = 1.

Electrochemical Measurements

All electrochemical tests were carried out on a CHI 760e electrochemical workstation (Chenhua Instruments Co., Ltd.) coupled with a rotating-ring-disk electrode (DC-DSRROTATOR, PHYCHEMI) in a three-electrode system. Ag/AgCl (3 M KCl) and platinum foil (1 cm × 1 cm) were used as the reference and counter electrode, respectively. The inks of the electrocatalysts were obtained by dispersing 4 mg electrocatalysts, 2 mg Ketjen Black and 5 μl Nafion (5 wt%) in 1ml ethanol, followed by sonication for 30 min. Then, 4 μl electrocatalyst ink were drop-coated and dried on the glassy carbon electrode (GCE, diameter: 5 mm) to form a thin film, which was used as the working electrode.

Methanol oxidation reaction (MOR) of all electrocatalysts were conducted in N_2-saturated aqueous electrolyte at room temperature. The working electrode was electrochemically activated in 0.1 M $HClO_4$ between -0.2 V and 1.0 V for 100 cycles at 200 mV s^{-1}. After that, CVs were performed in N_2-saturated 0.1

M $HClO_4$ solution at a scan rate of 50 mV s^{-1}, and the electrochemical active surface areas (ECSAs) was estimated by measuring the charge associated with H_{upd} adsorption (Q_H) and the mass of catalyst on the electrode surface (M_{Pt}). Then, the specific ECSA was calculated based on the following equation:

$$ECSA = \frac{Q_H}{210\, uC/cm^2 \cdot M_{Pt}}$$

The CVs for MOR was conducted in N_2-saturated 0.1 M $HClO_4$ + 0.5 M CH_3OH solution between -0.2-1 V vs. Ag/AgCl at a scan rate of 50 mV s^{-1}. For the MOR stability tests, 5000 CV cycles were measured in 0.1 M $HClO_4$ + 0.5 M CH_3OH solution between -0.2-1 V vs. Ag/AgCl with a scan rate of 200 mV s^{-1}. After that, the electrocatalysts on GCE were collected for TEM measurement to study the micromorphology stability. CO stripping voltammetry was measured in 0.1 M $HClO_4$ to study the CO anti-poisoning ability. Before the tests, 0.1 M $HClO_4$ was first de-aerated with high-purity N_2. Then, CO was bubbled into the cell for 15 min while the potential of the working electrode was held at a constant potential of 0.1 V vs. RHE. Then N_2 was bubbled into the system for 15 min to remove CO gas. After that, CO stripping curves were recorded between 0.4-1.2 V vs. RHE at a scan rate of 20 mV s^{-1}. The peak area was determined using the baseline CV, and a normalization factor of 420 μg cm^{-2} was applied to calculate the ECSA.

MEA fabrication and single-cell tests.

Anode catalyst inks were prepared by dispersing e-PtNiCo, Nafion® solution and isopropyl alcohol, and then ultrasonically dispersed for 1 h. The active area of 5 cm^2 was obtained by spraying inks on Nafion® 115 membrane through ultrasonic spraying. The catalyst loading of both anode (e-PtNiCo) and cathode (JM Pt/C, 40 wt.%) was guaranteed to be 1 $mg_{Pt}\cdot cm^{-2}$. Three different anode catalysts were investigated in this study: e-PtNiCo, PtNiCo and commercial JM Pt/C (40 wt.%). The membrane electrode assemblies (MEAs) were fabricated by inserting the membrane between the anode and the cathode gas diffusion layer (YLS-30T,

SuZhou Sinero Technology Co., Ltd) through a hot-pressing process at 75 kgf, 130°C for 3 min. The fuel cell test system (Scribner 850, Scribner Associates Inc.) was used to test a single-cell. The flow rates of methanol solution (1 M) and humidified oxygen were 2 ml min^{-1} and 300 sccm. The stability curves were obtained at a constant current of 100 $mA \cdot cm^{-2}$ for 50 h at 65°C.

Computational Methods

Spin-polarized DFT calculations were performed using the PBE[1] functional and the projector augmented wave (PAW)[2, 3] potential as implemented in the Vienna Ab Initio Simulation Package (VASP)[4, 5]. The van der Waals interaction was corrected based on the DFT-D3 scheme[6]. An energy cutoff of 400 eV and a convergence criterion of 10^{-5} eV for self-consistent calculations was adopted. All structures were fully relaxed until the total force on each atom was less than 0.05 eV $Å^{-1}$. The thickness of the vacuum layer was larger than 15 Å. The PtNiCo and e-PtNiCo/TiN slab was modeled based on $2\sqrt{3} \times 2\sqrt{3} \times 1$ TiN (1 1 1) supercell and $2 \times 2 \times 1$ PtNiCo (1 1 1) supercell for our catalyst modeling. A Γ-centered $3 \times 3 \times 1$ k-point was used. VASPKIT code[7] and VESTA software[8] were used for calculation pre-processing and post-processing.

The adsorption Gibbs free energy of CO molecule on the surface was defined as $\Delta G_{ads\text{-}CO} = \Delta E_{ads\text{-}CO} + \Delta ZEP - T\Delta S$, where ΔE, ΔZEP, and ΔS are adsorption energy of CO molecule calculated by DFT, the correction of zero-point energy, and entropy difference, respectively. The $\Delta E_{ads\text{-}CO}$ is defined as $\Delta E_{ads\text{-}CO} = E_{system} - E_{slab} - E_{CO}$, where the E_{system}, E_{slab}, and E_{CO} are the DFT energy of the catalyst system with one CO molecule adsorbed on the surface, the catalyst system without CO adsorption, and one CO molecule, respectively.

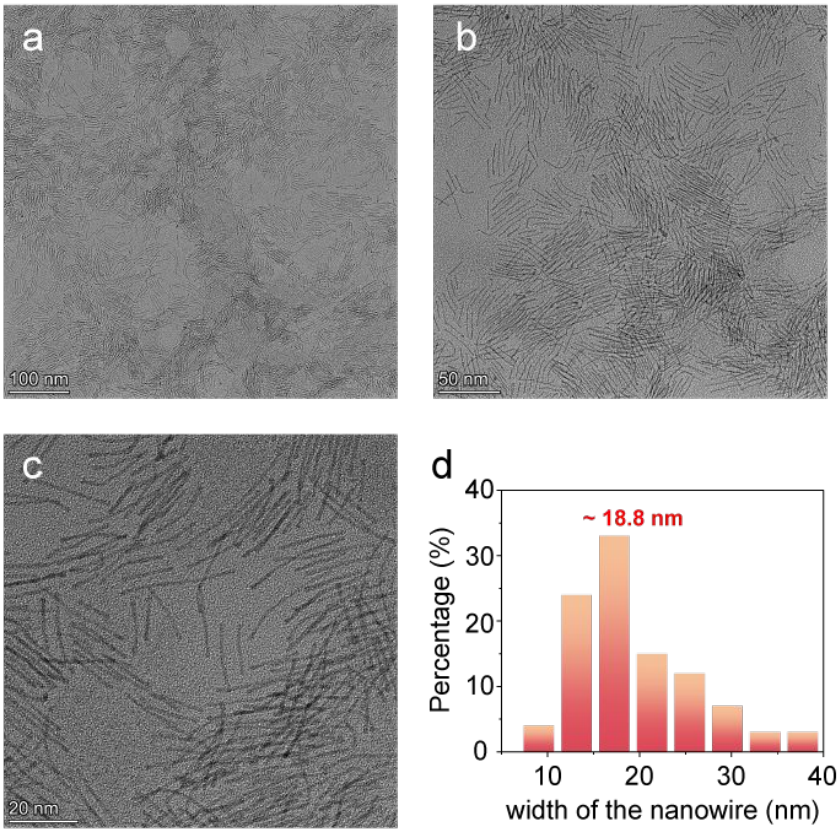

Figure S1. (a-c) TEM images and (d) the length distribution pattern of PtNiCo NWs.

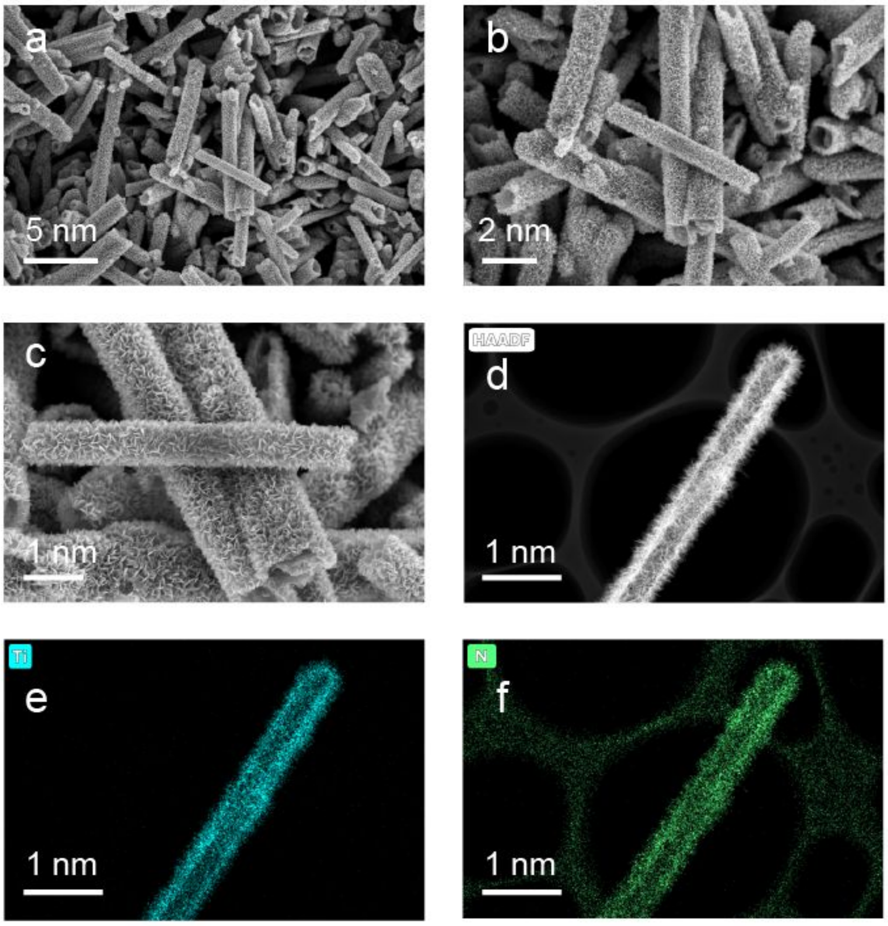

Figure S2. (a-c) SEM images and (d-f) TEM-EDS mapping of TiN nanotubes.

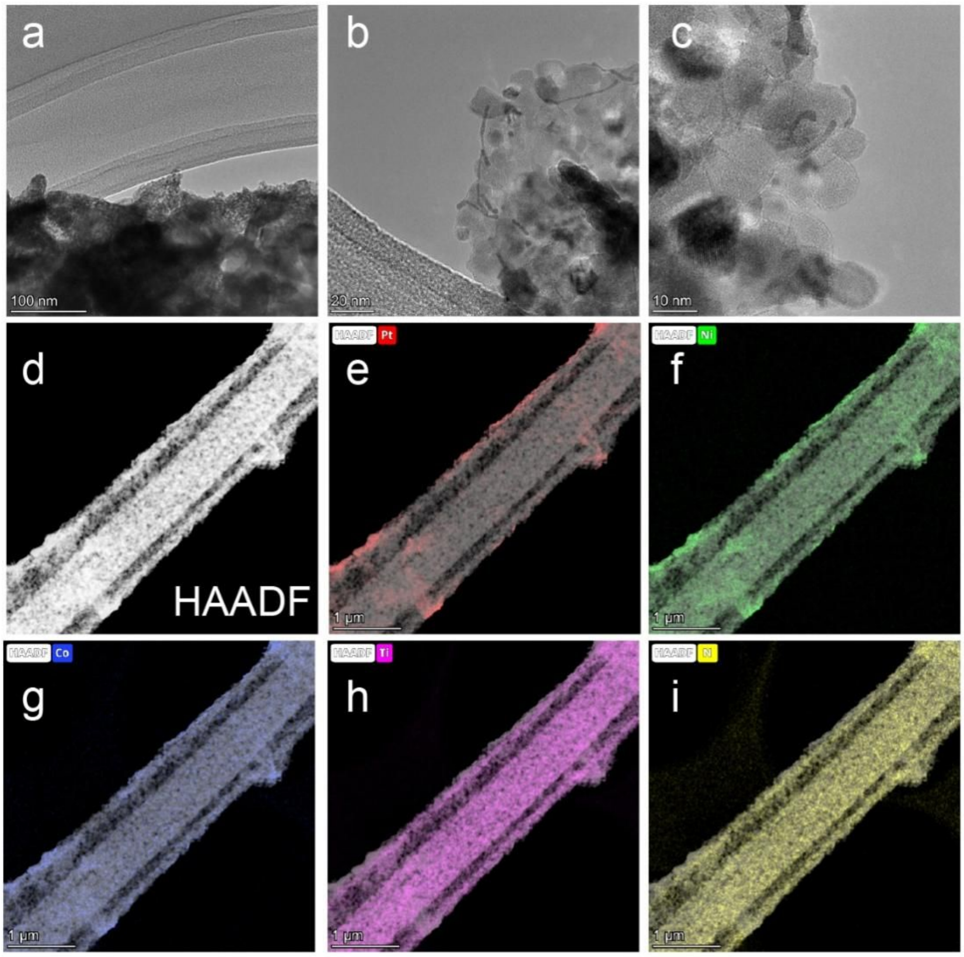

Figure S3. (a-c) TEM images and (d-i) TEM-EDS mapping of e-PtNiCo.

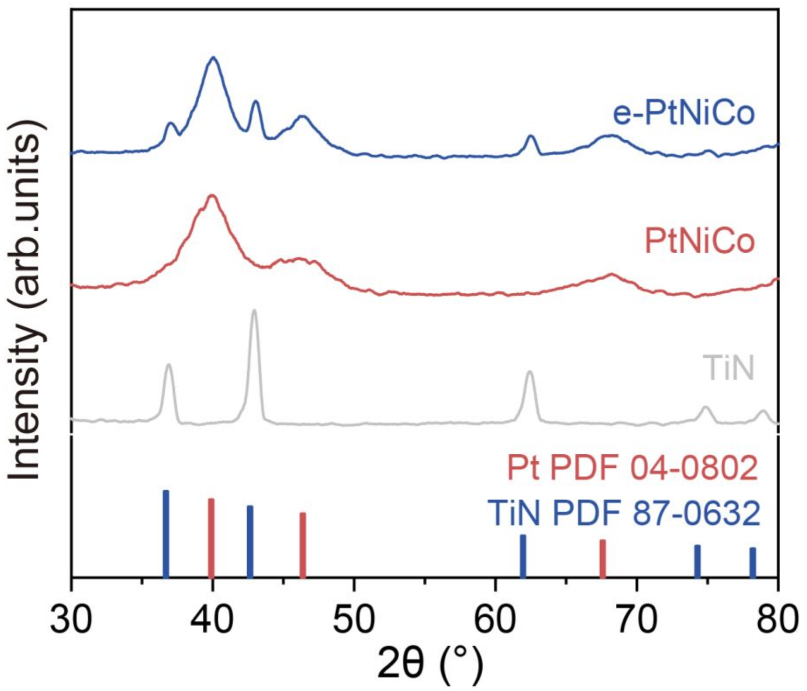

Figure S4. Powder XRD pattern of the prepared catalysts.

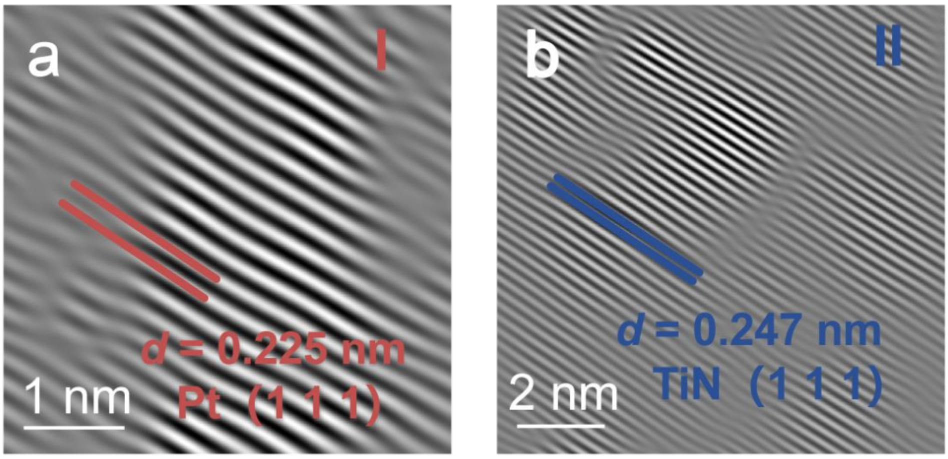

Figure S5. The corresponding lattice stripe spacing image obtained from the (a) Line I and (b) line II.

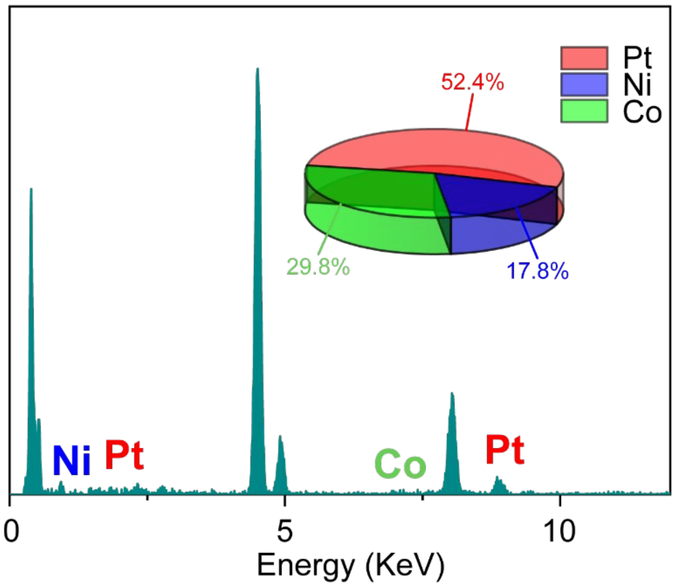

Figure S6. TEM-EDS spectrum of e-PtNiCo.

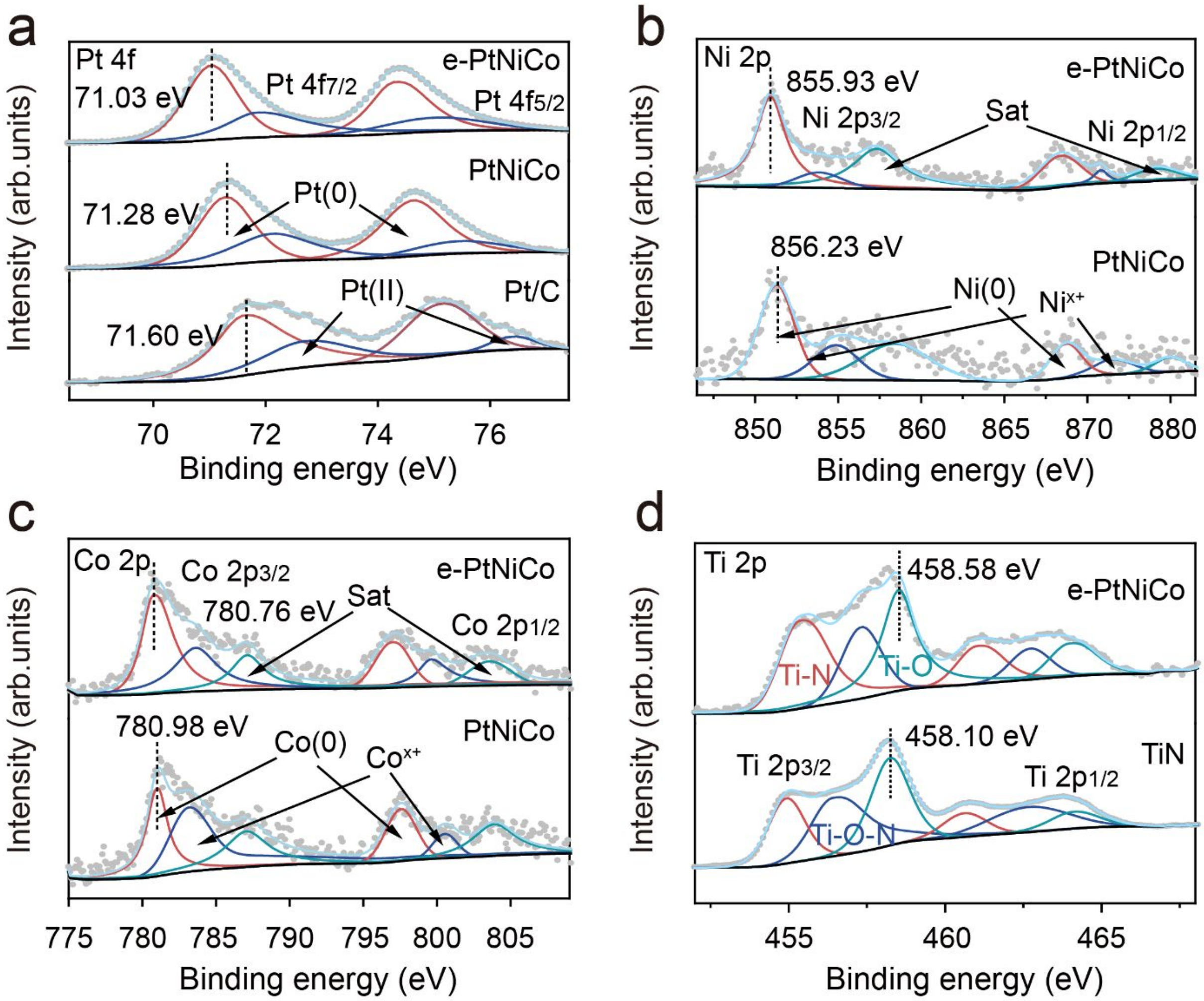

Figure S7. High-resolution XPS spectra of (a) Pt 4*f* of e-PtNiCo, PtNiCo and Pt/C, (b) Ni 2*p* and (c) Co 2*p* of e-PtNiCo and PtNiCo, (d) Ti 2*p* of e-PtNiCo and TiN.

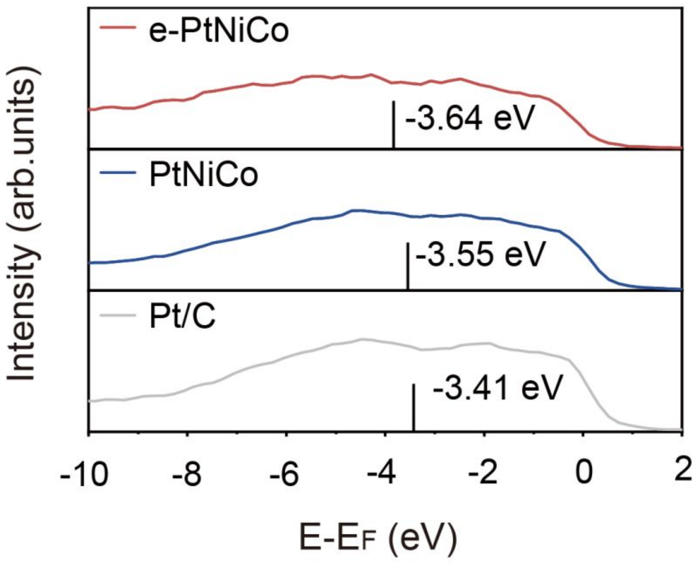

Figure S8. Valence band XPS spectra of e-PtNiCo, PtNiCo and Pt/C catalysts.

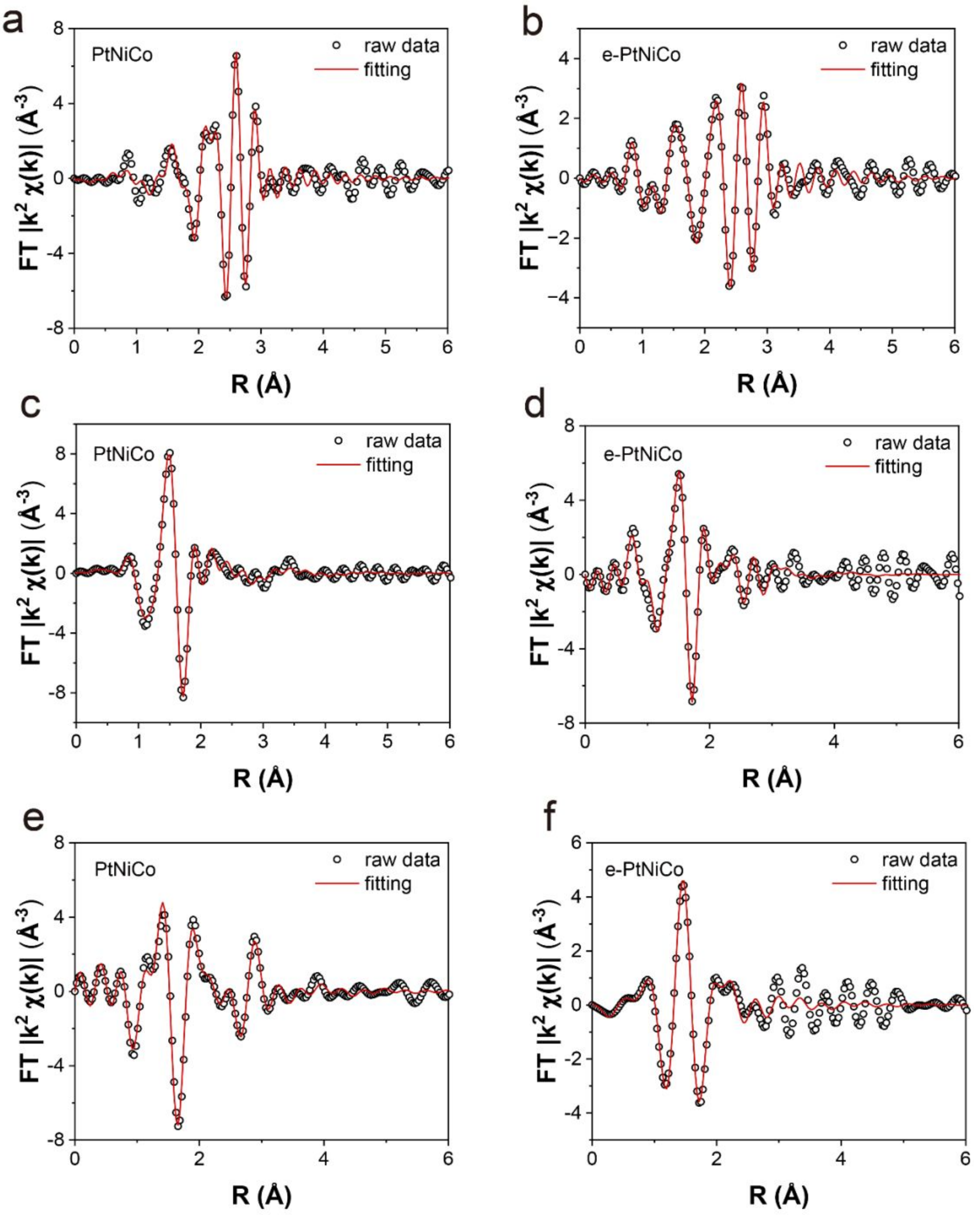

Figure S9. Imaginary part of EXAFS Fourier transformed results for PtNiCo and e-PtNiCo at (a-b) Pt L_3-edge (c-d) Ni *K*-edge, and (e-f) Co *K*-edge.

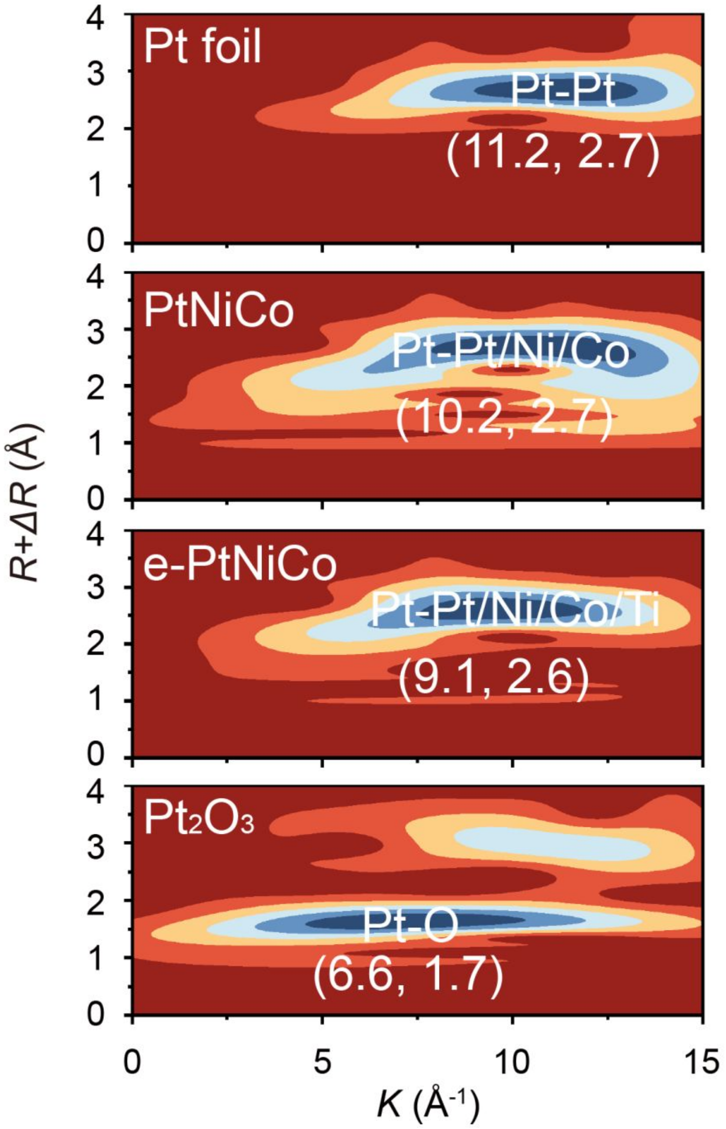

Figure S10. WT-EXAFS curves of e-PtNiCo, PtNiCo and standard samples.

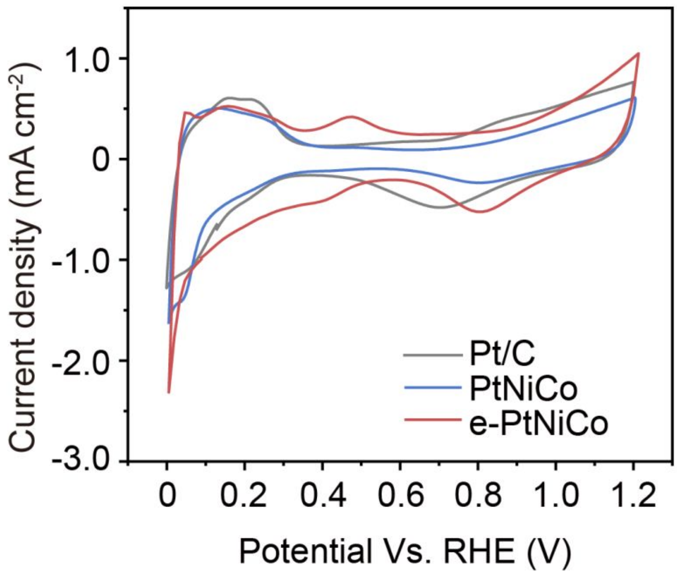

Figure S11. CVs of the prepared and commercial catalysts in N_2-saturated 0.1 M $HClO_4$ solution.

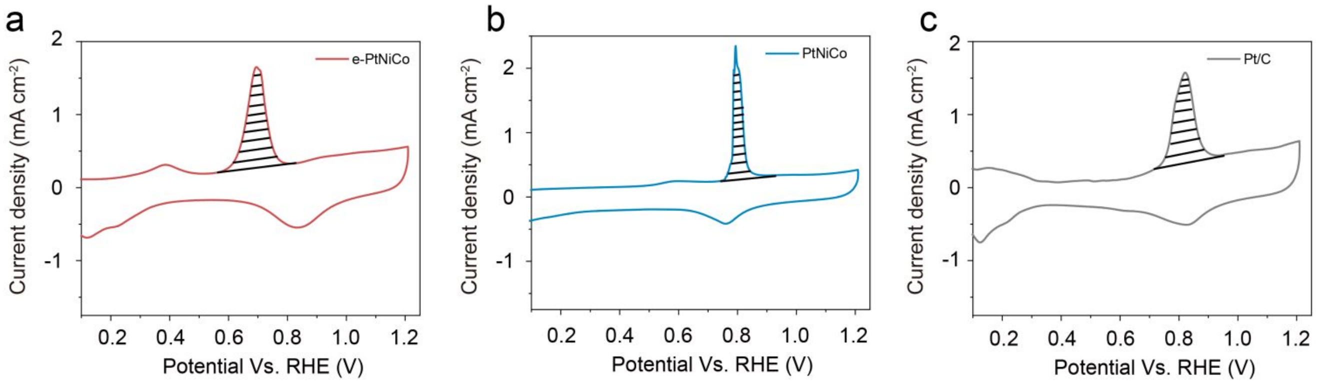

Figure S12. CO-stripping voltammetry curves of (a) e-PtNiCo, (b) PtNiCo, and (c) Pt/C.

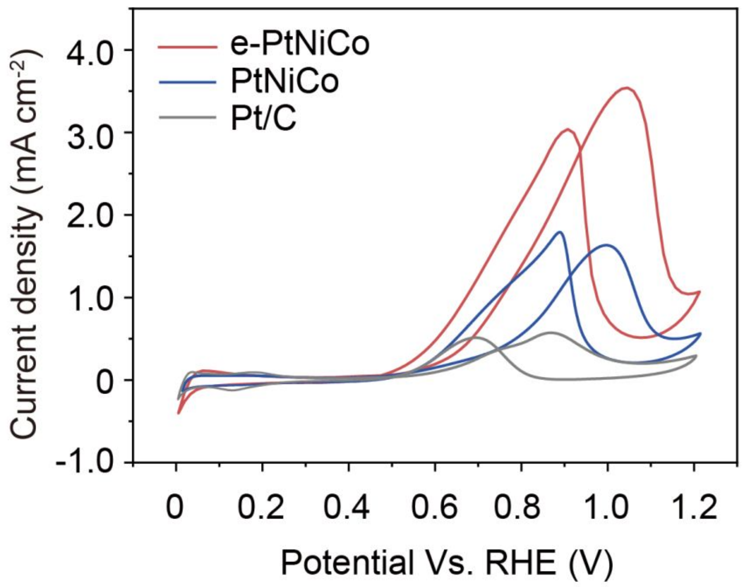

Figure S13. Specific activity of e-PtNiCo and reference samples in 0.1 M $HClO_4$ containing 0.5 M CH_3OH.

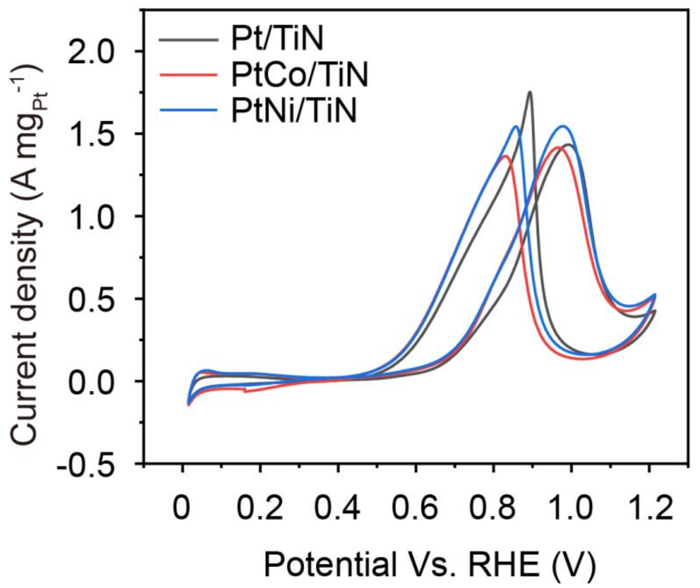

Figure S14. Mass activity of PtNi/TiN, PtCo/TiN and Pt/TiN catalysts in 0.1 M $HClO_4$ containing 0.5 M CH_3OH.

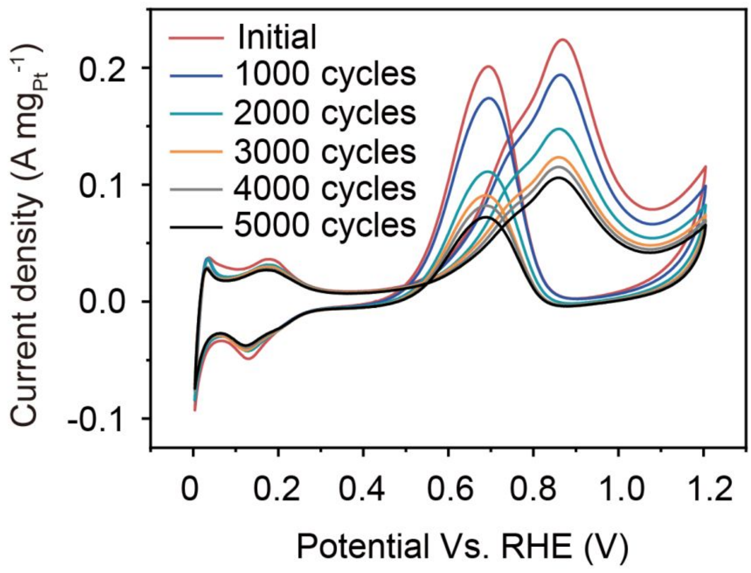

Figure S15. The evolution of CVs for Pt/C with continuous 5000 cycles ADT.

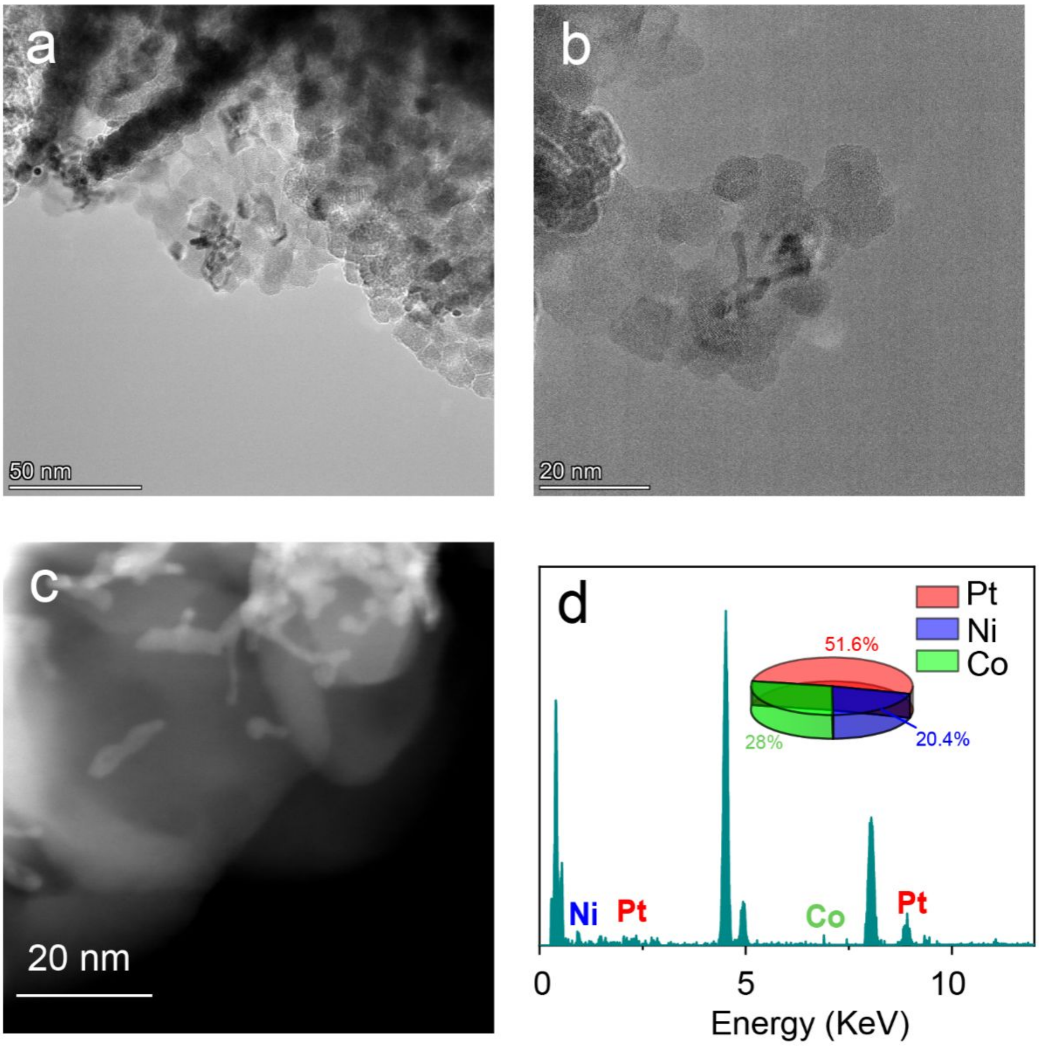

Figure S16. (a-b) TEM, (c) HRTEM images and (d) EDS spectrum of e-PtNiCo after ADT.

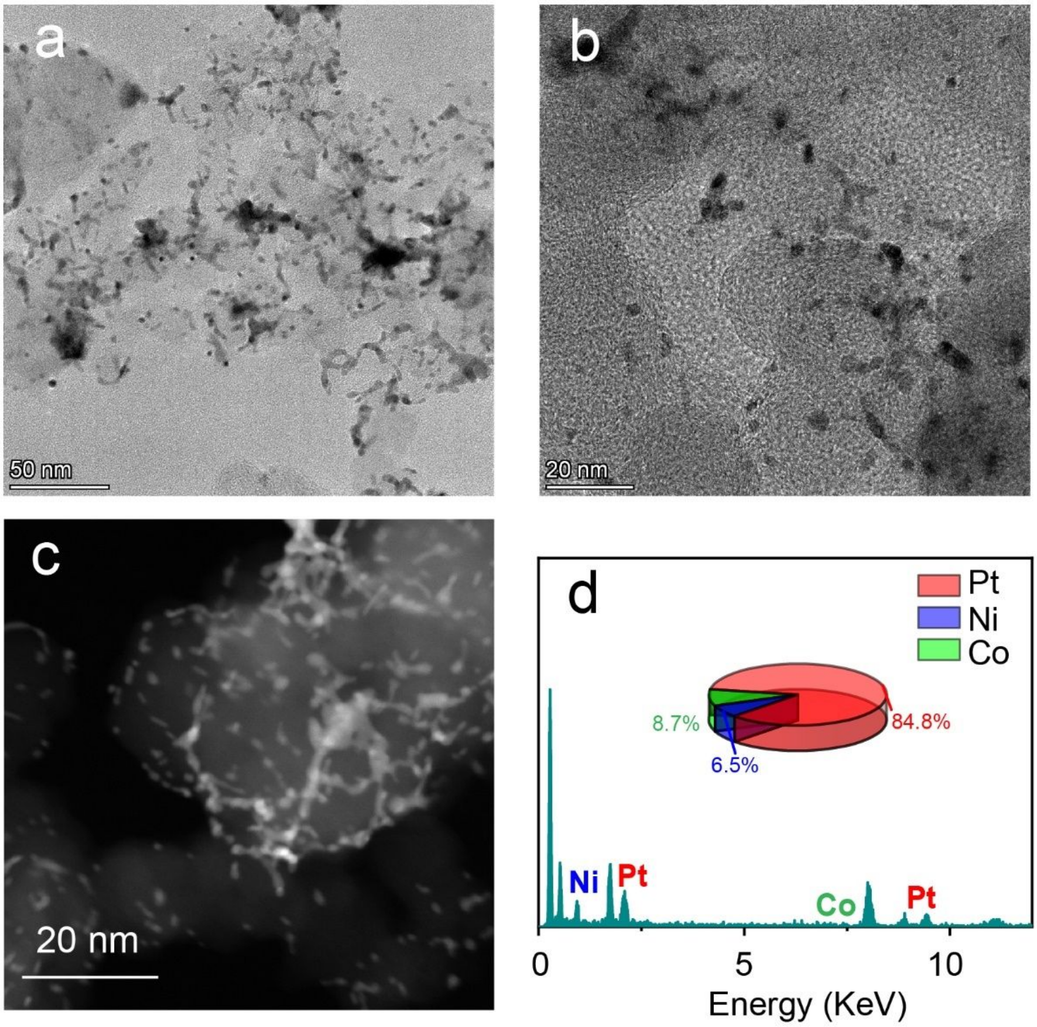

Figure S17. (a-b) TEM, (c) HRTEM images and (d) EDS spectrum of PtNiCo after ADT.

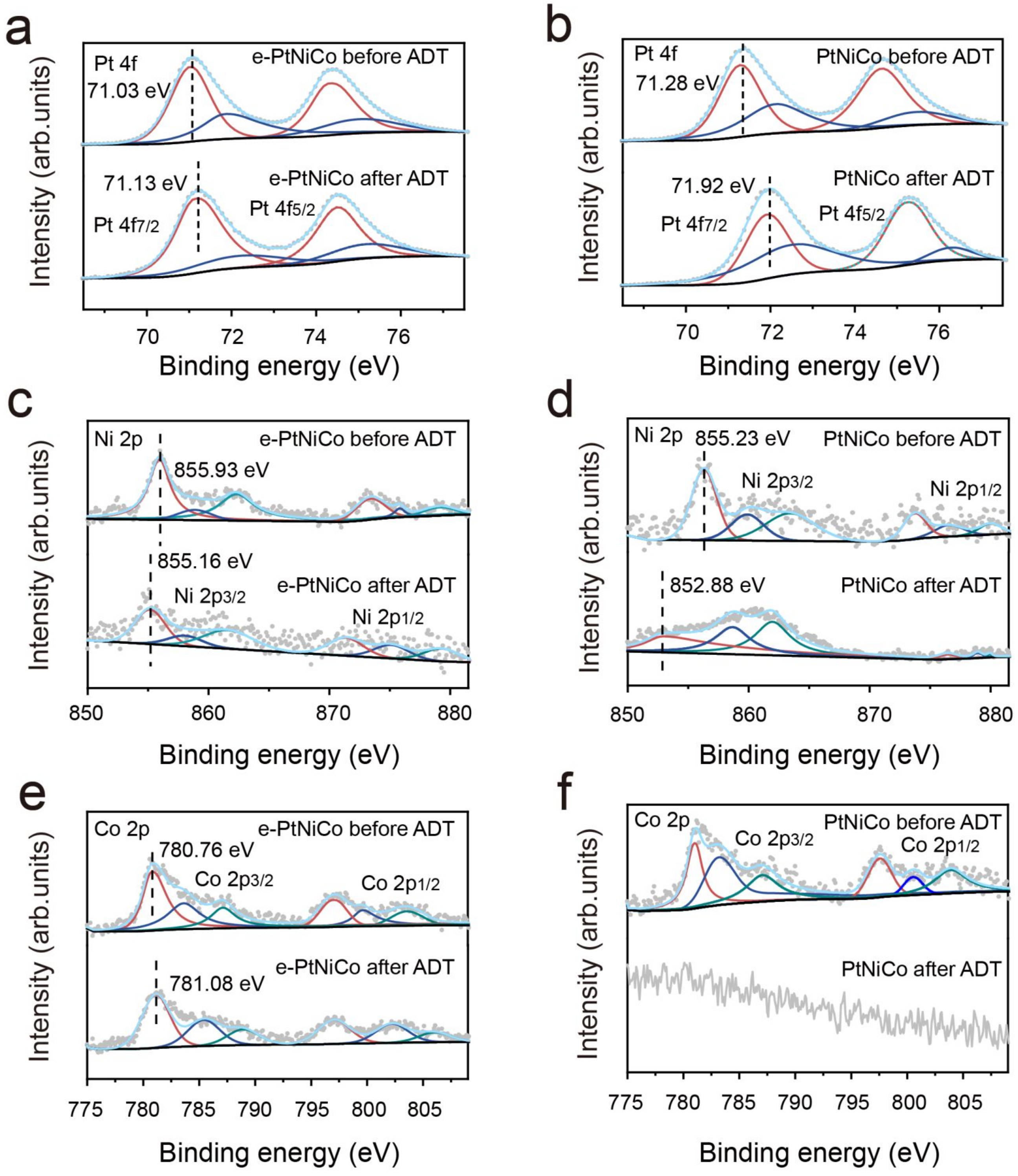

Figure S18. High-resolution XPS spectra of (a-b) Pt 4*f*, (c-d) Ni 2*p* and (e-f) Co 2*p* of e-PtNiCo and PtNiCo catalyst before and after ADT.

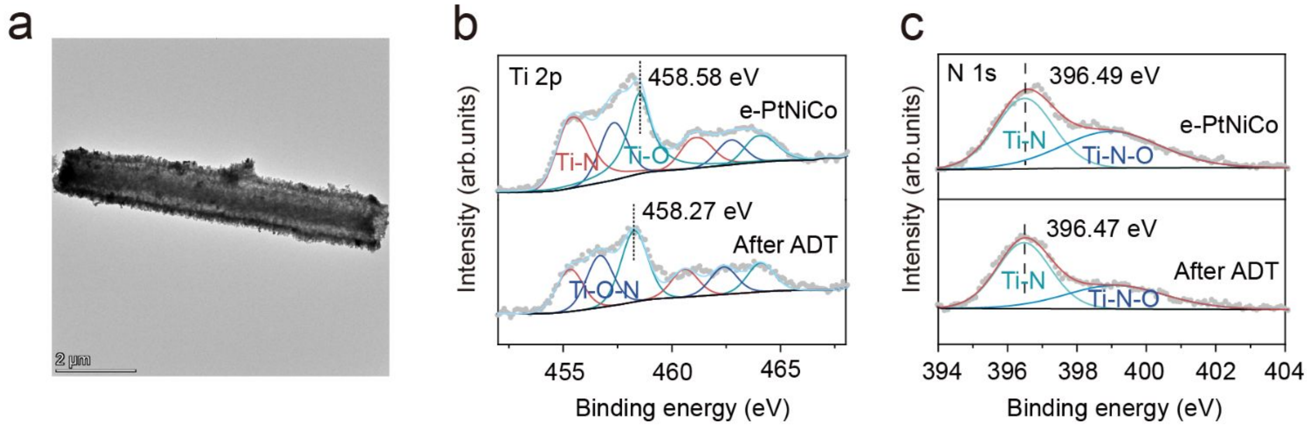

Figure S19. (a) TEM image of the e-PtNiCo catalyst after ADT. (b, c) High-resolution XPS spectra of Ti 2*p* and N 1*s* for the e-PtNiCo catalyst before and after ADT.

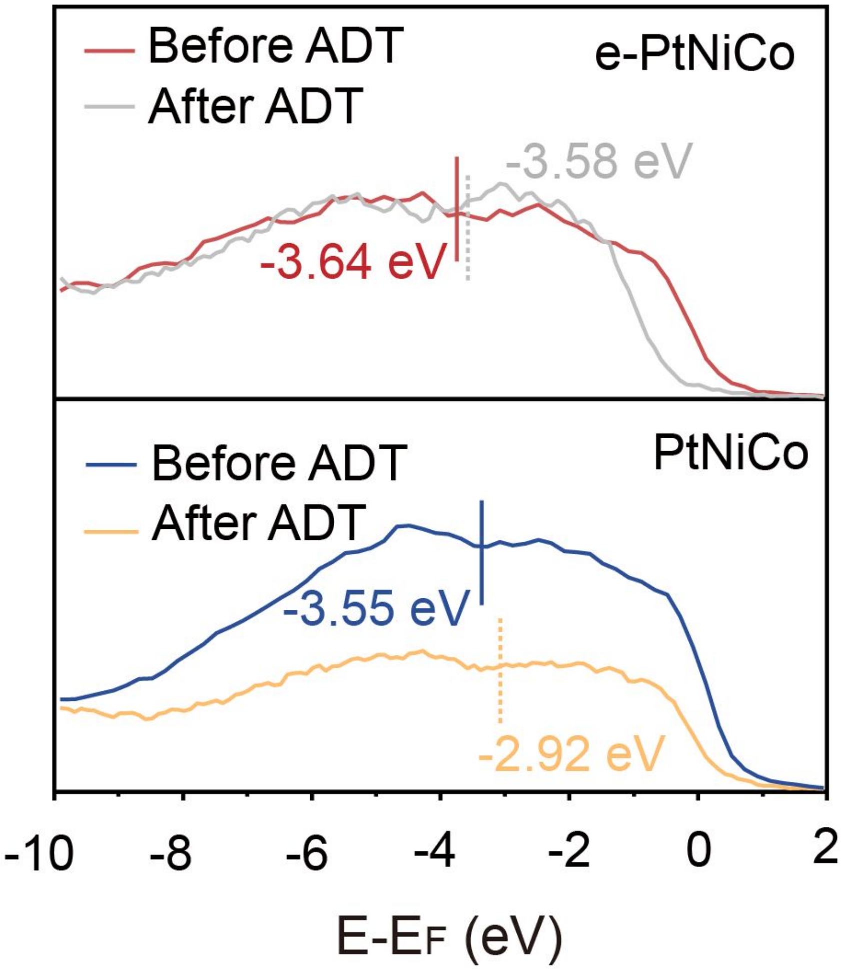

Figure S20. Valence band XPS spectra of e-PtNiCo and PtNiCo before and after ADT.

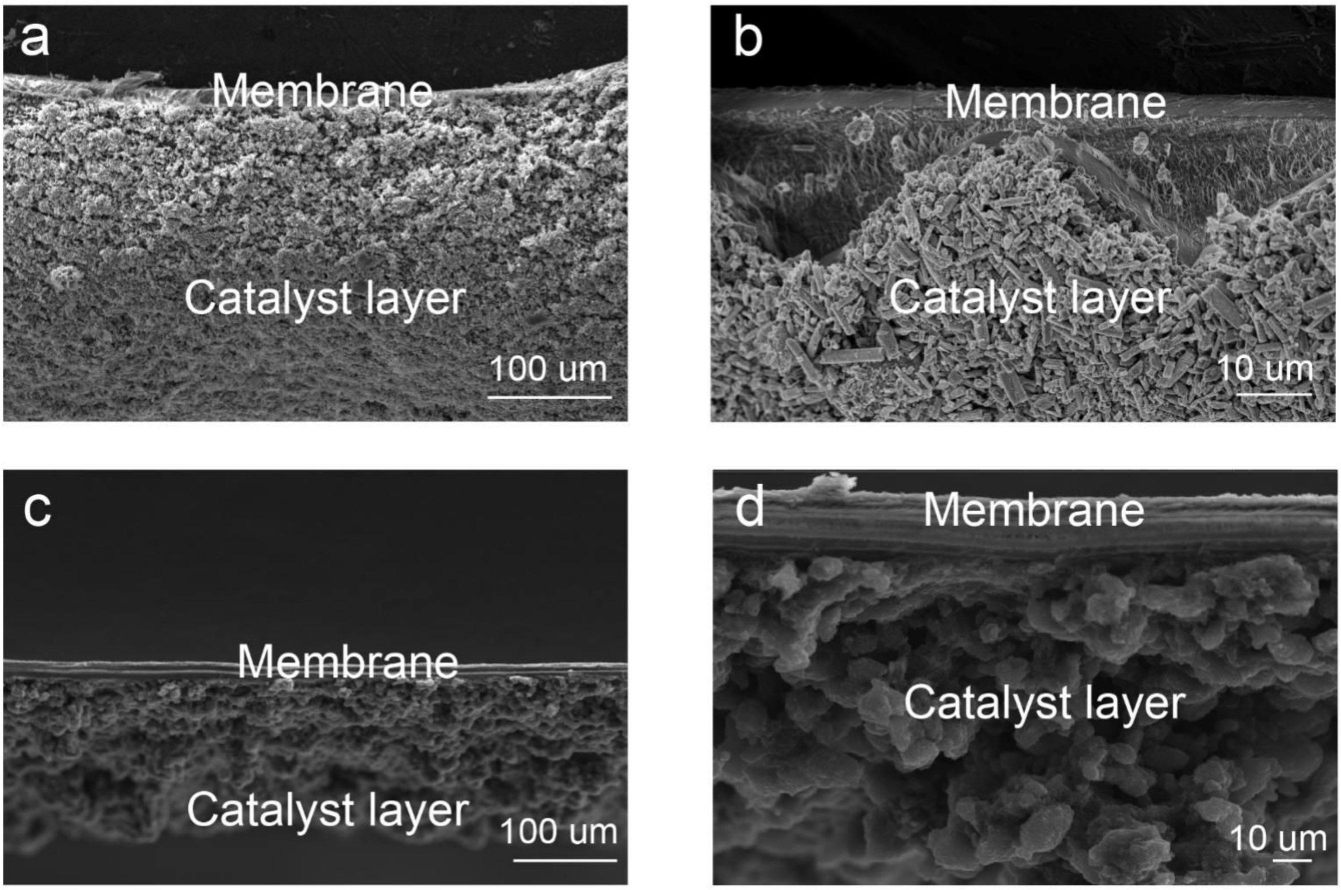

Figure S21. SEM of catalysts layer of (a-b) e-PtNiCo and (c-d) PtNiCo after 50 h constant current test.

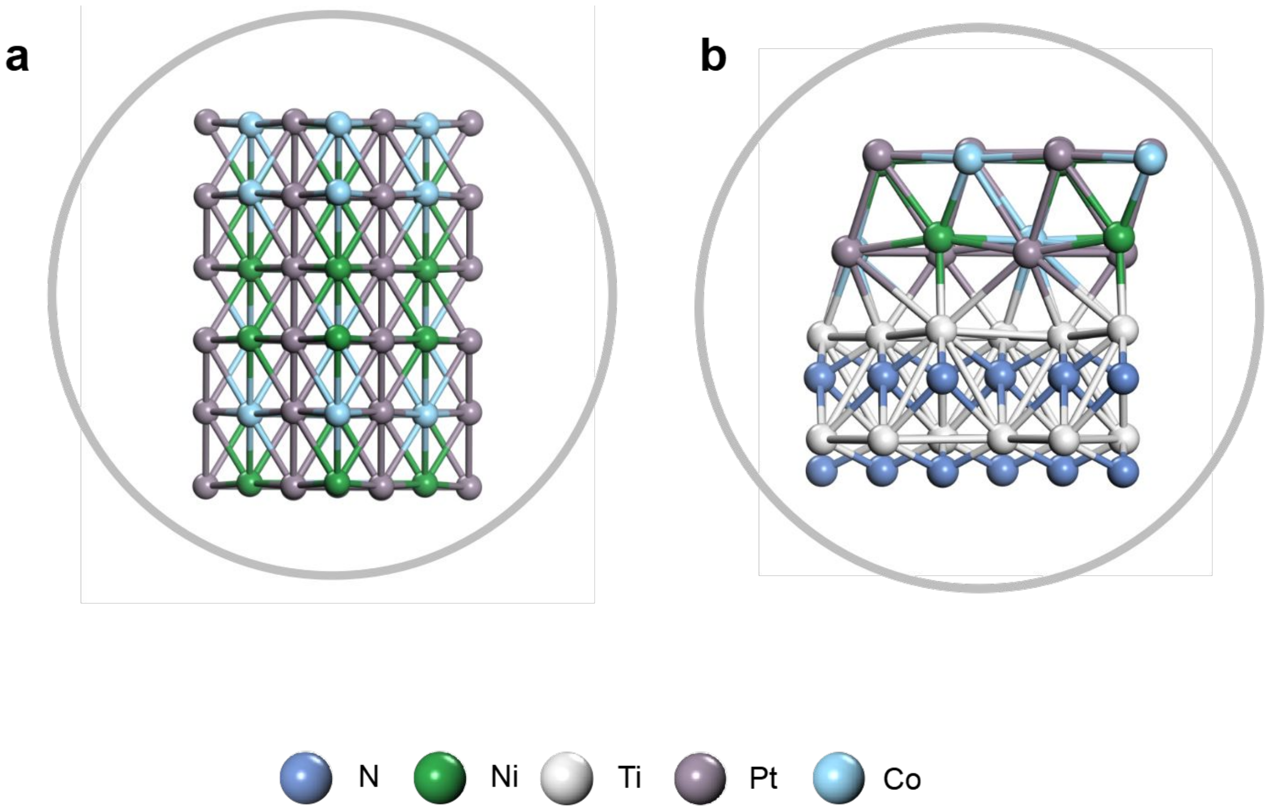

Figure S22. Atomic structure of (a) PtNiCo and (b) e-PtNiCo/TiN model.

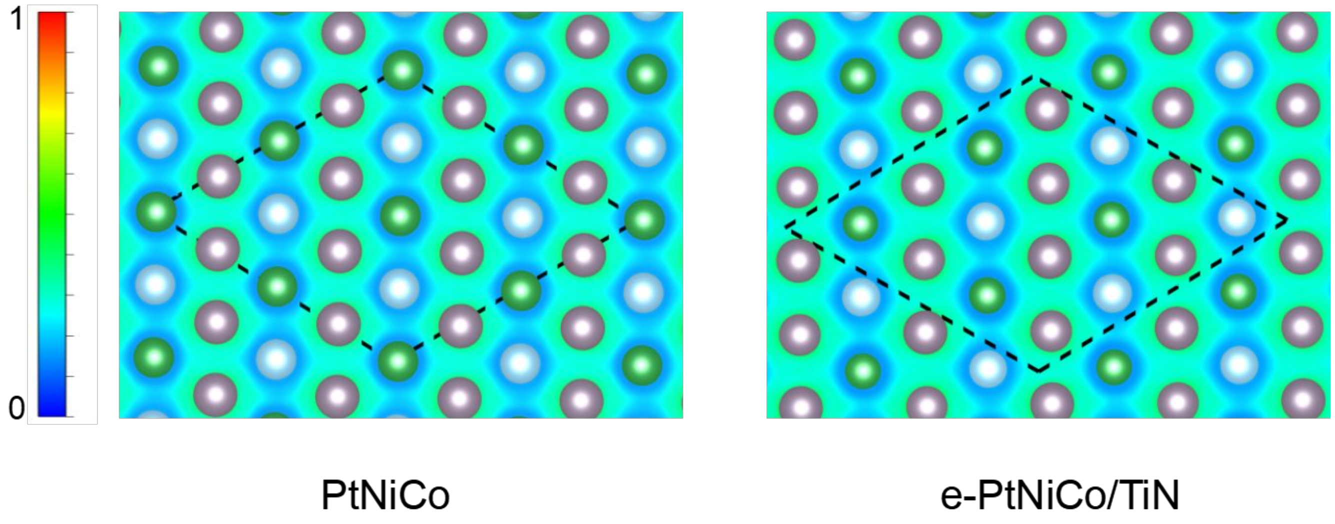

Figure S23. Calculated electron localization functions for PtNiCo and e-PtNiCo/TiN.

Table S1. ICP-MS analysis of catalysts.

ICP	e-PtNiCo	PtNiCo	Pt/C
Pt wt.%	20.48	20.73	20
Ni wt%	10.83	10.14	/
Co wt.%	11.62	12.23	/

Table S2. The results of Pt 4*f* peak fitting for e-PtNiCo, PtNiCo, and Pt/C catalysts.

Catalysts	Pt (0)		Pt (II)		$Pt^0/(Pt^2+Pt^0)$
	Binding energy (eV)	Ratio	Binding energy (eV)	Ratio	Ratio
e-PtNiCo	71.03	49%	71.87	51%	49%
PtNiCo	71.28	42%	72.18	58%	42%
Pt/C	71.60	39%	72.70	61%	39%

Table S3. EXAFS data fitting results of e-PtNiCo and PtNiCo.

Catalyst	Path	CN	R (Å)	σ^2 (Å^{-2})	ΔE_0 (eV)	R factor
e-PtNiCo (Pt)	Pt-N	2.0	2.01±0.025	0.019±0.001	2.6±0.5	0.007
	Pt-Pt	5.4	2.71±0.020	0.009±0.006		
	Pt-Ni	2.3	2.67±0.040	0.019±0.008		
	Pt-Co	2.3	2.50±0.026	0.019±0.008		
	Pt-Ti	0.6	2.64±0.023	0.009±0.006		
PtNiCo (Pt)	Pt-C	2.0	2.08±0.027	0.021±0.002	3.77±1.47	0.017
	Pt-Pt	5.4	2.73±0.007	0.006±0.001		
	Pt-Ni	2.3	2.72±0.059	0.021±0.003		
	Pt-Co	2.3	2.52±0.042	0.021±0.003		
e-PtNiCo (Ni)	Ni-N	6	2.10±0.014	0.004±0.001	-3.00±1.73	0.017
	Ni-Pt/Ti	2.3	2.64±0.003	0.006±0.003		
	Ni-Ni	3.2	2.21±0.050	0.035±0.009		
PtNiCo (Ni)	Ni-C	5.6	2.09±0.011	0.006±0.001	-4.36±0.75	0.008
	Ni-Pt	2.3	2.65±0.008	0.026±0.017		
	Ni-Ni	3.2	2.22±0.039	0.029±0.007		
e-PtNiCo (Co)	Co-N	3.2	2.01±0.009	0.003±0.001	-6.00±1.8	0.008
	Co-Pt	2.3	2.58±0.033	0.024±0.005		
	Co-Co/Ti	3.2	2.55±0.033	0.024±0.005		
PtNiCo (Co)	Co-C	6	2.08±0.007	0.013±0.001	9.9±1.6	0.010
	Co-Pt	2.3	2.59±0.026	0.012±0.003		
	Co-Co	3.2	2.58±0.027	0.011±0.003		

CN: coordination numbers; R: bond distance; σ^2: Debye-Waller factors; ΔE_0: the inner potential correction. R factor: goodness of fit. $S0_2$ was fixed as 1.0, which were obtained from the experimental EXAFS fit of reference by fixing CN as the known crystallographic value and was fixed to all the samples. Data ranges of e-PtNiCo (Pt) and PtNiCo (Pt) were $2.0 \leqslant k \leqslant 11$ Å^{-1} and $1.0 \leqslant R \leqslant 3.0$ Å, data ranges of e-PtNiCo (Ni) and

PtNiCo (Ni) were 3.0≤k≤12 Å^{-1} and 1.0≤R≤3.0 Å and data ranges of e-PtNiCo (Co) and PtNiCo (Co) were 3.0≤k≤9.0 Å^{-1} and 1.0≤R≤3.0 Å.

Table S4 The comparison of performance of e-PtNiCo, PtNiCo, and Pt/C for MOR in 0.1 M $HClO_4$ containing 0.5 M CH_3OH solution.

	ECSA ($m^2\ g_{Pt}^{-1}$)	MA ($mA\ mg_{Pt}^{-1}$)	SA ($mA\ cm^{-2}$)
e-PtNiCo	59.4	1735	3.54
PtNiCo	58.1	790	1.63
Pt/C	57.8	279	0.58

Table S5 Comparison of MOR activity and stability with previously reported catalysts under acidic media.

Catalysts	Methanol concentration (M)	Scan rate (mV s^{-1})	Specific activity (mA cm^{-2})	Mass activity (mA mg_{Pt}^{-1})	Ref.
e-PtNiCo	0.5	50	3.54	1735	This work
PtCu/Ce-CuO_x/C	0.5	50	0.92	333	[9]
PtCu/$Pr_{0.15}Ce_{0.85}O_2$	0.5	50	3.67	1060	[10]
Pt/H-TiN/NC	1	50	1.15	1346	[11]
Pt/C@TiO_2	0.5	50	0.9	590	[12]
Pt/$Ti_{0.7}Cu_{0.3}N$	0.5	50	1.48	840	[13]
Pt/3D $Ti_{0.75}Mo_{0.25}N$	0.5	50	1.63	980	[14]
UE-NOMWCNTs/Pt	1	20	0.48	607	[15]
Pt/LP-TiO_2/CFP	1	50	0.51	1183	[16]
PtNi100/TNTs/Ti	1	50	0.78	410	[17]
Pt-TiO_2-rNHGO	1	50	0.68	591	[18]
Pt1/ $Ti_{0.8}W_{0.2}N_xO_y$	0.5	50	0.46	560	[19]

Pt_3Sn@u-SnO_2/NG	1	50	1.86	1482	20
PtRu/TiO_2/ONCNT	0.5	50	0.77	512	21
UV-Pt@TiO_2/GN	1	0.5	3.21	1945	22
Pt-Ag DSNCs	1	50	3.18	567	23
p-Pt/TNR@GC	0.5	50	2.48	1120	24
PtNi/CeO_x	0.5	50	4.07	1500	25
Pt/Fe_3O_4-SNG	0.5	50	2.46	1129	26

Table S6 Comparison of DMFCs activity and stability with previously reported catalysts.

Anode catalyst	Pt loading	MeOH (M)	T (℃)	OCV (V)	P_{max} (mW cm^{-2})	M_{max} (W g^{-1})	Stability (activity retention%)	Ref.
e-PtNiCo	1 mg_{Pt} cm^{-2}	1	65	0.66	107.4	107.4	90.4%, 50 h, at 100 mA cm^{-2}	This work
PtNiCo	1 mg_{Pt} cm^{-2}	1	65	0.64	50.2	50.2	62.3%, 25 h, at 100 mA cm^{-2}	
Pt/C	1 mg_{Pt} cm^{-2}	1	65	0.55	39.6	39.6	58.1%, 25 h, at 100 mA cm^{-2}	
p-Pt@p-NCNT	1 mg_{Pt} cm^{-2}	1	65	0.65	65.8	65.8	89.6%, 500 h, at 100 mA cm^{-2}	27
PtRu/C	2.5 mg cm^{-2}	1	65	0.80	/	/	~87.5%, 180 h, at 100 mA cm^{-2}	28
PtPd NCs/C	3 mg cm^{-2}	10	70	~0.81	232.0	77.3	83.8%, 24 h, at 200 mA cm^{-2}	29
Pt-Ni_2P/C	1 mg_{Pt} cm^{-2}	1	60	~0.50	65.0	65	~95.0%, 24 h, at 0.3 V	30
Pt-CoP/C	1.2 mg_{Pt} cm^{-2}	2	70	~0.60	88.5	73.7	~93.0%, 12 h, at 0.3 V	31
rGO/PANI/Pt-Pd	2 mg_{Pt} cm^{-2}	1	70	~0.81	117.5	58.7	82.0%, 70 h, OCV	32
Pt-$NiTiO_3$/C	0.5 mg_{Pt} cm^{-2}	1	80	~0.43	32.8	65.7	78.0%, 5 h, at 100 mA cm^{-2}	33
Pt/$Ce_{0.7}Mo_{0.3}O_{2-\delta}$-C	2 mg_{Pt} cm^{-2}	1	60	~0.98	69.4	69.4	/	34
PtRu NPs	4 mg cm^{-2}	2	80	~0.60	68.7	17.2	/	35
PtRu/C	2 mg cm^{-2}	2	70	0.80	55.9	28.0	87.9%, 10000 s, at 200 mA cm^{-2}	36
PtRu/C	2 mg cm^{-2}	1	90	0.75	163.0	81.5	78.0%, 66 h, at 100 mA cm^{-2}	37

References

1. Perdew, J. P., et al., Generalized Gradient Approximation Made Simple. *Phys. Rev. Lett.* **1996,** *77*, 3865-3868.

2. Blöchl, P. E., Projector Augmented-Wave Method. *PRB* **1994,** *50*, 17953-17979.

3. Kresse, G.; Joubert, D., From Ultrasoft Pseudopotentials to the Projector Augmented-Wave Method. *PRB* **1999,** *59*, 1758-1775.

4. Kresse, G.; Furthmüller, J., Efficient Iterative Schemes for Ab Initio Total-Energy Calculations Using a Plane-Wave Basis Set. *PRB* **1996,** *54*, 11169-11186.

5. Kresse, G.; Furthmüller, J., Efficiency of Ab-Initio Total Energy Calculations for Metals and Semiconductors Using a Plane-Wave Basis Set. *Comp. Mater. Sci.* **1996,** *6*, 15-50.

6. Grimme, S., et al., A Consistent and Accurate Ab Initio Parametrization of Density Functional Dispersion Correction (DFT-D) for the 94 Elements H-Pu. *J. Chem. Phys.* **2010,** *132*, 154104.

7. Wang, V., et al., Vaspkit: A User-Friendly Interface Facilitating High-Throughput Computing and Analysis Using Vasp Code. *Comput. Phys. Commun.* **2021,** *267*, 108033.

8. Momma, K.; Izumi, F., Vesta 3 for Three-Dimensional Visualization of Crystal, Volumetric and Morphology Data. *J. Appl. Crystallogr.* **2011,** *44*, 1272-1276.

9. Wang, Y., et al., Renovating Phase Constitution and Construction of Pt Nanocubes for Electrocatalysis of Methanol Oxidation via a Solvothermal-Induced Strong Metal-Support Interaction. *Appl. Catal. B Environ.* **2023,** *325*, 122383.

10. Zou, T., et al., Defect-Engineered Charge Transfer in a PtCu/$Pr_xCe_{1-x}O_2$ Carbon-Free Catalyst for Promoting the Methanol Oxidation and Oxygen Reduction Reactions. *ACS Appl. Mater. Interfaces* **2023,** *15*, 58296-58308.

11. Tang, M., et al., Pt Nanoparticles Supported on N-Doped Carbon/Mesoporous TiN Particle Composites as Catalysts for the Methanol Oxidation Reaction. *ACS Appl. Nano Mater.* **2024,** *7*, 1606-1614.

12. Zhang, W., et al., TiO_2 Nanolayer-Coated Carbon as Pt Support for Enhanced Methanol Oxidation Reaction. *J. Electrochem. Energy.* **2024,** *21*, 041006.

13. Liu, F., et al., Platinum-Decorated Three Dimensional Titanium Copper Nitride Architectures with Durable Methanol Oxidation Reaction Activity. *Int. J. Hydrogen Energy* **2019,** *44*, 8415-8424.

14. Liu, F., et al., Three Dimensional Titanium Molybdenum Nitride Nanowire Assemblies as Highly Efficient and Durable Platinum Support for Methanol Oxidation Reaction. *Electrochim. Acta* **2019,** *295*, 50-57.

15. Li, W., et al., Stepwise Preparation of Uniform-Size Ultrafine Pt Nanoparticles for High-Performance Catalysis of Methanol Oxidation and Nitrophenol Reduction. *ACS Appl. Nano Mater.* **2023,** *6*, 19176-19188.

16. Deng, J., et al., Fabrication of Layered Porous TiO_2/Carbon Fiber Paper Decorated by Pt Nanoparticles Using Atomic Layer Deposition for Efficient Methanol Electro-Oxidation. *J. Electroanal. Chem.* **2020,** *874*, 114468.

17. Chang, Y., et al., One-Step Synthesis of PtNi Anchored on TiO_2 Nanotube Arrays for Methanol Oxidation. *J. Alloy. Compd.* **2023,** *943*, 169179.

18. Cui, R., et al., N-Doping Holey Graphene TiO_2-Pt Composite as Efficient Electrocatalyst for Methanol Oxidation. *ACS Appl. Energy Mater.* **2020,** *3*, 2665-2673.

19. Awoke, Y. A., et al., The Synergistic Effect Pt_1-W Dual Sites as a Highly Active and Durable Catalyst for Electrochemical Methanol Oxidation. *Electrochim. Acta* **2022,** *432*,

141161.

20. Wang, Z., et al., Pt_3Sn Nanoparticles Enriched with SnO_2/Pt_3Sn Interfaces for Highly Efficient Alcohol Electrooxidation. *Nanoscale Adv* **2021,** *3*, 5062-5067.

21. Sekar, A., et al., PtRu Catalysts on Nitrogen-Doped Carbon Nanotubes with Conformal Hydrogenated TiO_2 Shells for Methanol Oxidation. *ACS Appl. Nano Mater.* **2022,** *5*, 3275-3288.

22. Zhang, K., et al., Morphological Tuning Engineering of $Pt@TiO_2$/Graphene Catalysts with Optimal Active Surfaces of Support for Boosting Catalytic Performance for Methanol Oxidation. *J. Mater. Chem. A* **2022,** *10*, 4254-4265.

23. Yao, W., et al., Engineering Hollow Porous Platinum-Silver Double-Shelled Nanocages for Efficient Electro-Oxidation of Methanol. *Appl. Catal. B Environ.* **2021,** *282*, 119595.

24. Li, C., et al., Synthesis of Well-Defined Pt-Based Catalysts for Methanol Oxidation Reaction Based on Electron-Hole Separation Effects. *ACS Sustainable Chem. Eng.* **2019,** *7*, 8597-8603.

25. Kwon, Y., et al., One-Pot Production of Ceria Nanosheet-Supported PtNi Alloy Nanodendrites with High Catalytic Performance toward Methanol Oxidation and Oxygen Reduction. *J. Mater. Chem. A* **2020,** *8*, 25842-25849.

26. Zhong, J., et al., Worm-Like Pt Nanoparticles Anchored on Graphene with S, N Co-Doping and Fe_3O_4 Functionalization for Boosting the Electrooxidation of Methanol. *Int. J. Hydrogen Energy* **2020,** *45*, 22929-22937.

27. Hu, T., et al., Plasma-Induced Formation of Pt Nanoparticles with Optimized Surface Oxidation States for Methanol Oxidation and Oxygen Reduction Reactions to Achieve High-Performance DMFCs. *Small* **2023,** *15*, 2304076.

28. Li, W., et al., Liquid-Solid Heterogeneous Synthesis of Highly Dispersed and Pdpt Surface Enriched PdPtCu/C as Methanol Tolerant Oxygen Reduction Reaction Catalysts. *Appl. Catal. B Environ.* **2013,** *129*, 426-436.

29. Li, W., et al., In Situ Shaped PtPd Nanocubes on Common Carbon Powder for Efficient Methanol Electrooxidation in Practical Fuel Cells. *Int. J. Hydrogen Energy* **2024,** *50*, 1496-1506.

30. Chang, J., et al., Ni_2P Enhances the Activity and Durability of the Pt Anode Catalyst in Direct Methanol Fuel Cells. *Energ. Environ. Sci.* **2014,** *7*, 1628-1632.

31. Chang, J., et al., Pt-CoP/C as an Alternative PtRu/C Catalyst for Direct Methanol Fuel Cells. *J. Mater. Chem. A* **2016,** *4*, 18607-18613.

32. Arukula, R., et al., Cumulative Effect of Bimetallic Alloy, Conductive Polymer and Graphene toward Electrooxidation of Methanol: An Efficient Anode Catalyst for Direct Methanol Fuel Cells. *J. Alloy. Compd.* **2019,** *771*, 477-488.

33. Kumaresan, T., et al., Effect of Nafion Loading and the Novel Flow Field Designs on Innovative Anode Electrocatalyst for Improved Direct Methanol Fuel Cells Performance. *Mater. Lett.* **2020,** *276*, 128222.

34. Zhang, G., et al., Nanosized Mo-Doped CeO_2 Enhances the Electrocatalytic Properties of the Pt Anode Catalyst in Direct Methanol Fuel Cells. *J. Mater. Chem. A* **2017,** *5*, 1481-1487.

35. Li, Y., et al., Self-Healing Proton-Exchange Membranes Composed of Nafion-Poly (Vinyl Alcohol) Complexes for Durable Direct Methanol Fuel Cells. *Adv. Mater.* **2018,** *30*, 1707146.

36. Wang, W., et al., Self-Standing CoFe Embedded Nitrogen-Doped Carbon Nanotubes with Pt Deposition through Direct Current Plasma Magnetron Sputtering for Direct Methanol Fuel Cells Applications. *Carbon* **2023,** *201*, 1068-1080.

37. Qi, J., et al., Improving the Activity and Stability of a Pt/C Electrocatalyst for Direct Methanol Fuel Cells. *Carbon* **2010,** *48*, 163-169.
